# Bone-Targeting Nanoparticles of a Dendritic (Aspartic acid)_3_-Functionalized PEG-PLGA Biopolymer Encapsulating Simvastatin for the Treatment of Osteoporosis in Rat Models

**DOI:** 10.3390/ijms231810530

**Published:** 2022-09-11

**Authors:** Che-Wei Lin, Chih-Yun Lee, Sung-Yen Lin, Lin Kang, Yin-Chih Fu, Chung-Hwan Chen, Chih-Kuang Wang

**Affiliations:** 1 Department of Medicinal and Applied Chemistry, College of Life Science, Kaohsiung Medical University, Kaohsiung 80708, Taiwan; 2 Regenerative Medicine and Cell Therapy Research Center, Office of Research and Development, Kaohsiung Medical University, Kaohsiung 80708, Taiwan; 3 Orthopaedic Research Center, College of Medicine, Kaohsiung Medical University, Kaohsiung 80708, Taiwan; 4 Ph.D. Program in Life Sciences, College of Life Science, Kaohsiung Medical University, Kaohsiung 80708, Taiwan; 5 Departments of Orthopaedics, School of Medicine, College of Medicine, Kaohsiung Medical University, Kaohsiung 80708, Taiwan; 6 Department of Orthopaedics, Kaohsiung Medical University Hospital, Kaohsiung Medical University, Kaohsiung 80708, Taiwan; 7 Department of Orthopaedics, Kaohsiung Municipal Ta-Tung Hospital, Kaohsiung Medical University, Kaohsiung 80145, Taiwan; 8 Department of Obstetrics and Gynecology, National Cheng Kung University Hospital, College of Medicine, National Cheng Kung University, Tainan 70101, Taiwan; 9 Ph.D. Program in Biomedical Engineering, College of Medicine, Kaohsiung Medical University, Kaohsiung 80708, Taiwan; 10 Graduate Institute of Medicine, College of Medicine, Kaohsiung Medical University, Kaohsiung 80708, Taiwan

**Keywords:** bone targeting, nanoparticles, aspartic acid, simvastatin, osteoporosis, disuse

## Abstract

Simvastatin (SIM) is a lipid-lowering drug that also promotes bone formation, but its high liver specificity may cause muscle damage, and the low solubility of lipophilic drugs limits the systemic administration of SIM, especially in osteoporosis (OP) studies. In this study, we utilized the bone-targeting moiety of dendritic oligopeptides consisting of three aspartic acid moieties (_d_Asp_3_) and amphiphilic polymers (poly(ethylene glycol)-block-poly(lactic-co-glycolic acid); PEG-PLGA) to create _d_Asp_3_-PEG-PLGA (APP) nanoparticles (NPs), which can carry SIM to treat OP. An in vivo imaging system showed that gold nanocluster (GNC)-PLGA/APP NPs had a significantly higher accumulation rate in representative bone tissues. In vivo experiments comparing low-dose SIM treatment (0.25 mg/kg per time, 2 times per week) showed that bone-targeting SIM/APP NPs could increase the bone formation effect compared with non-bone-targeting SIM/PP NPs in a local bone loss of hindlimb suspension (disuse) model, but did not demonstrate good bone formation in a postmenopausal (ovariectomized) model of systemic bone loss. The APP NPs could effectively target high mineral levels in bone tissue and were expected to reduce side effects in other organs affected by SIM. However, in vivo OP model testing showed that the same lower dose could not be used to treat different types of OP.

## 1. Introduction

Osteoporosis (OP) is a major public health concern, especially considering the aging population worldwide, and is a progressive skeletal disease characterized by a decrease in bone mass and microarchitecture deterioration of bone tissue, often leading to different levels of deterioration at different skeletal sites [1]. Therefore, OP typically results in decreased bone strength and a higher probability of fractures [2]. The most frequently affected sites of osteoporotic injury are the hip, spine, and wrist [3,4,5,6]. Bone is a living tissue that is broken down and replaced every day. OP occurs when the creation of new bone cannot keep up with the loss of old bone [7,8]. The two main types of OP that are common in adults are primary and secondary OP. Primary OP is either age-related or has an unknown cause [7,9]. In addition, primary OP can be divided into two types. Type 1, which is also known as postmenopausal OP, generally develops in women after menopause due to decreases in estrogen production. However, it can also occur due to testosterone deficiency. This type is more common in women than in men and typically occurs at the ages of 50–70 years [9]. Type 2, which is also known as senile OP, typically develops after the age of 70 in older men and women, although it is more common in women [9]. Secondary OP occurs when the loss of bone is caused by certain medical conditions, such as steroids, which affect bone mass and cause bone loss or other health problems [10,11]. In addition, disuse (immobilization-induced) is one of the many reasons for bone loss and results in secondary OP [10,12]. Disuse OP is a common skeletal disorder in elderly individuals, in patients subjected to prolonged immobility or bed rest, e.g., fracture and spinal injury, and in astronauts who participate in long-duration spaceflight [13].

Currently, available therapies for the treatment of OP include exercise, calcium supplementation, postmenopausal estrogen therapy, calcitonin therapy, and the administration of bisphosphonates, parathyroid hormone (PTH), or calcitriol [14]. Regardless of the approach used to treat OP, the main treatment methods currently involving drugs can be divided into two categories: one aims to inhibit bone resorption, and the second aims to enhance the effect on bone formation [15,16,17]. In this study, we focus on statins, which have the potential ability to promote new bone development, because many studies have mentioned that statins can increase bone mass by enhancing bone morphogenetic protein-2 (BMP-2)-mediated osteoblast expression in vitro and in animals [18,19,20,21]. Unfortunately, the negative effects and properties of statins on bones in the body, such as rhabdomyolysis, inflammatory myopathies, myasthenia gravis, Guillain Barre-like syndrome, tendinopathy, and amyotrophic lateral sclerosis (ALS) or ALS-like syndromes, limit their clinical application. Therefore, statins are beneficial for osteoporotic patients, and low statin oral bioavailability should be considered [22,23,24]. Although local delivery of simvastatin (SIM) is a more reliable and effective method for such bone healing purposes, systemic administration of SIM is also a valuable option in OP. Moreover, higher doses of SIM are generally required in the systemic delivery method, which increases drug side effects. The use of targeted drug delivery systems may help increase the bioavailability and distribution of statins to the bone microenvironment while also reducing the side effects. However, it is challenging to correlate statin delivery and distribution with the bone microenvironment at an effective concentration [25,26].

Although nanomedicine employing advanced nanotechnology has shown beneficial effects on the reversal of OP [27,28], the treatment of OP with statin drugs has been rarely explored, which may be due to the lack of a suitable statin bone-targeting carrier in current clinical OP treatments. Therefore, the development of an OP-targeted system might be challenging but is highly desirable for different OP models. For example, H. Wang et al. reported that SIM-loaded tetracycline-poly(lactic-co-glycolic acid) (PLGA) nanoparticles can deliver SIM to bone sites to treat ovariectomized (OVX) OP by promoting osteoblast differentiation and mineralization. However, the size of the tetracycline-PLGA nanoparticles was relatively large, and the drug-loading efficacy was not high. In addition, tetracycline has certain side effects [29,30].

Some studies in the past decade have reported that various types of acidic oligopeptides, such as glutamic acid (Glu)_n_, aspartic acid (Asp)_n_, and aspartic acid-serine-serine (Asp-Ser-Ser)_n_, have shown affinity at bone sites after intravenous injection [31,32,33]. The main reason is that carboxylic acids, such as Glu and Asp, have good biocompatibility, and they are rapidly degraded and removed from the plasma. Tao et al. published the synthesis of linear oligopeptides composed of six Asp molecules (ASP_6_) as bone-targeting moieties grafted to lipid nanoparticles (LNPs) to increase the concentration of SIM in bones at a relatively low dose to minimize the adverse effects [30]. Their results revealed that SIM/ASP_6_-LNP nanocarriers can promote targeted delivery of the anti-OP drug SIM in OVX rats [30]. However, questions remain about the dose and frequency of SIM administration since there are still no results describing the exact response in a real situation. For example, their study only discussed the OVX model and no other OP models [30].

**Figure 1:**
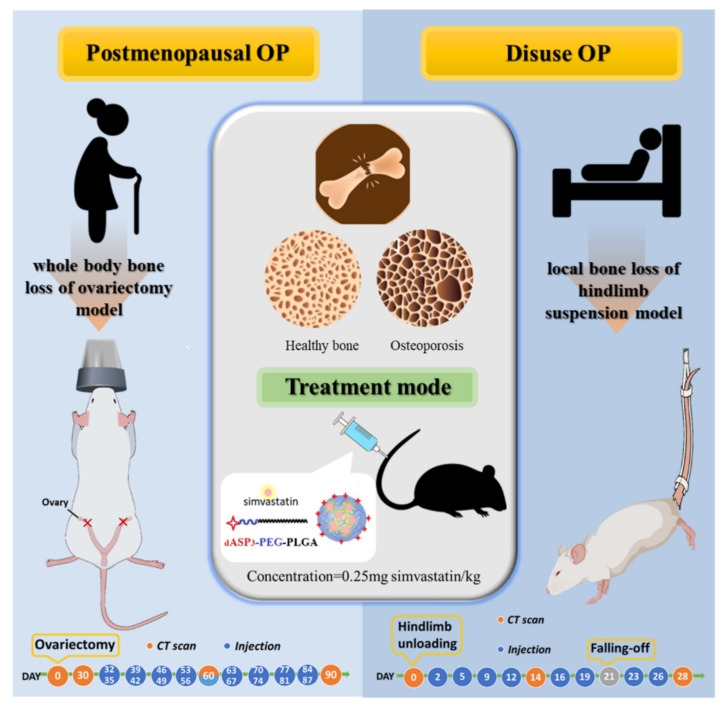
Scheme of the main research framework and objectives for the novel bone-targeting moiety of dendritic oligopeptide by three aspartic acids (_d_Asp_3_) and amphoteric polymer (poly(ethylene glycol-block-poly(lactic-co-glycolic acid); PEG-PLGA) to create _d_Asp_3_-PEG-PLGA (APP) nanoparticles (NPs) with bone-targeting capability, which will carry SIM to treat osteoporosis disease. Osteoporosis in the animal model will be classified into the primary type of postmenopausal model for systemic bone loss using the ovariectomized (OVX) method and the secondary type of disuse model for local bone loss using the hindlimb suspension method.

Biodegradable poly(hydroxyl acid) compounds, such as copolymers of poly(lactic acid) (PLA) and poly(lactic-co-glycolic acid (PLGA), are being used extensively in biomedical applications because of their biocompatibility [33,34,35], their ability to encapsulate various drug molecules, and their sustained release properties [34,35]. In addition, designed PLGA-PEG (PLGA-graft-polyethylene glycol) blocks copolymers to form nanoparticles. This results in decreased protein adsorption, apparently because of the shielding of the targeting moieties by longer PEG chains [35]. Notably, the bone-targeting delivery systems of _d_Asp_3_-PEG-PLGA (APP) nanoparticles (NPs) were developed using the esterification reaction for synthesis, and the average particle size was less than 200 nm. The dendritic oligopeptides obtained by using three aspartic acid (_d_Asp_3_) moieties of the APP NPs exhibited the best apatite mineral binding ability in our previous study [35]. The _d_Asp_3_ oligopeptide is negatively charged and able to bind calcium ions near the surface of hydroxyapatite. Additionally, the _d_Asp_3_ oligopeptide is not cytotoxic and can effectively promote the targeted delivery of bone tissue in vivo in zebrafish and rats [35]. We showed controlled-release SIM properties in which the amount of SIM released daily was within the concentration range of 0.1–5 μM/day, which significantly stimulated osteogenic gene expression of RUNX-2, BMP-2, alkaline phosphatase, osteocalcin, and final mineralization in cultured bone marrow stromal cells [36,37]. In vivo experiments have shown that the properties of controlled-release SIM not only enhance callus formation during the beginning of fracture healing but also increase the formation of new blood vessels and cell in-growth in the grafted bone [21,36,37]. Therefore, we used bone-targeting nanoparticles (NPs) of APP to carry SIM to form SIM-laden APP (SIM/APP) NPs and treat OP in animal models, as shown in Figure 1, which presents the research framework and objectives. SIM-laden non-bone-targeting PEG-PLGA (SIM/PP) NPs were used as a control. The physical and chemical properties of the biomaterials, the mechanism of the cell endocytosis pathway of the APP NPs, the SIM encapsulation efficacy of the NPs, and their mineralization ability in vitro were evaluated. We expected to obtain some evidence regarding treatment for specific OP models and to effectively reduce the SIM dose and frequency of use. In vivo experiments were classified into primary experiments, consisting of the postmenopausal model for systemic bone loss using the OVX method, and secondary experiments, consisting of the disuse model for local bone loss, using the hindlimb suspension method to evaluate treatment efficiency for these novel bone-targeting SIM/APP NPs and non-bone-targeting SIM/PP NPs.

## 2. Results and Discussion

### 2.1. Identification of _d_Asp_3_-PEG-PLGA and PEG-PLGA

Two types of amphiphilic block copolymers (_d_Asp_3_-PEG-PLGA and PEG-PLGA) were successfully synthesized according to our previous methods [35]. The bone-targeting functional amphiphilic block copolymer _d_Asp_3_-PEG-PLGA (APP) has a dendritic trimer aspartic acid group at the end of PEG that conjugates with PLGA. The related chemical evidence for each step, comprising the ^1^H nuclear magnetic resonance (NMR) spectrum, Fourier transform infrared spectroscopy (FTIR) spectrum, and electrospray ionization (ESI) mass spectrum, are presented in Figures S1–S3 in the Supplementary Materials.

### 2.2. Evaluation of GNCs and GNC Coupling with PLGA

**Figure 2:**
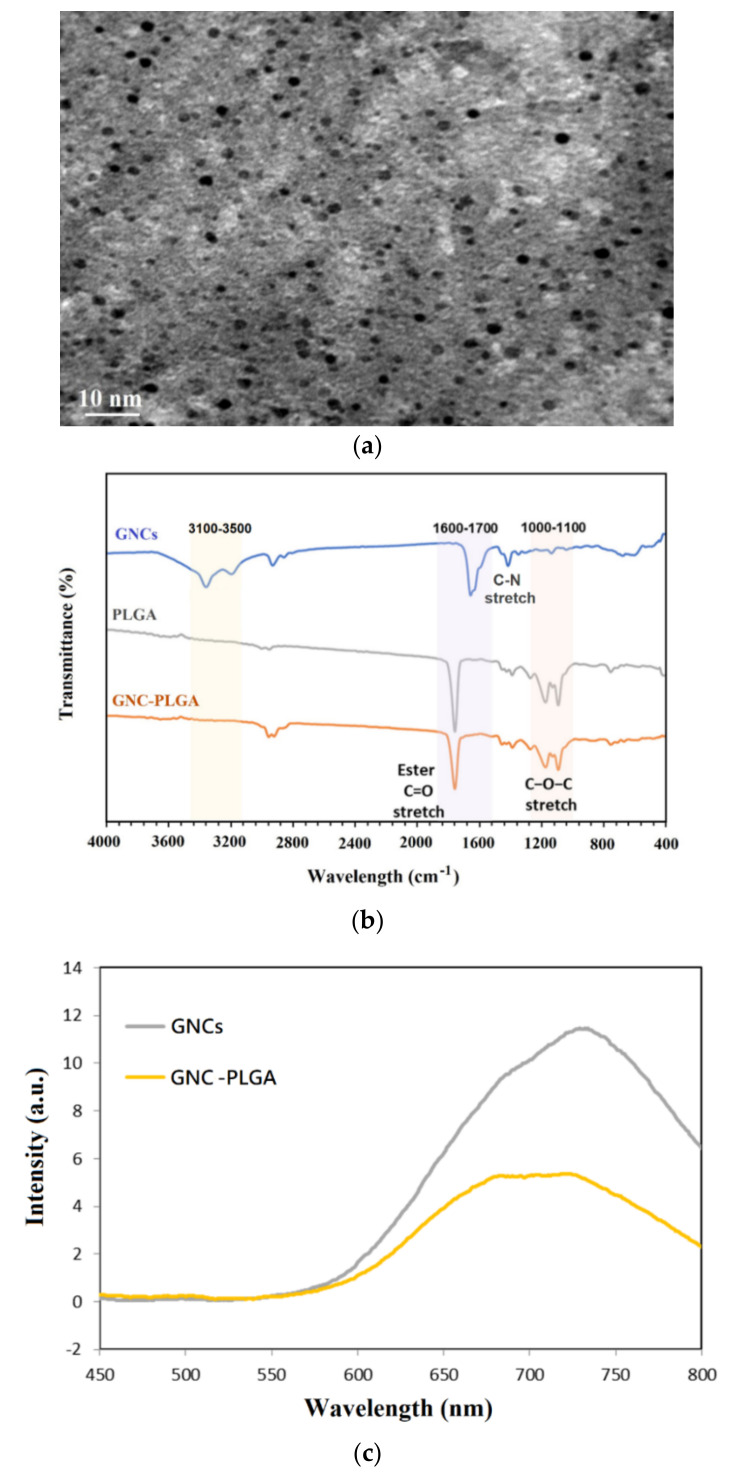
TEM images of fluorescent gold nanoclusters (GNCs) (**a**); FTIR spectra of GNCs, PLGA, and GNC-PLGA (**b**); fluorescence emission (excitation at 350 nm) spectra of GNC colloid and GNC-PLGA solution (**c**).

The morphology and size range of the GNCs were analyzed by TEM, and an image is shown in Figure 2a. The GNCs were nearly spherical, and the average GNC diameter was approximately 2–3 nm. The FTIR spectra of GNCs from 400 to 4000 cm^−^^1^ are shown in Figure 2b. Owing to the synthesis of GNCs coupled with lipoamide ligands (Figure S4 in the Supplementary Materials), the surface functional groups included a 1° amide (CONH_2_). However, the S-S stretching vibrations (671 cm^−1^ and 518 cm^−1^) of free lipoamide disappeared completely after coupling with GNCs, which is attributed to the S–Au interaction [38]. Some vibrational modes survived and were still observed in the FTIR spectrum of the coupled GNCs, with shifting of the spectral signals and variations in the dimensions, such as the amide I band of the GNC-lipoamide at 1600–1700 cm^−1^ (C=O stretching), the amide II band near 1380–1500 cm^−1^ (C-N stretching coupled with NH bending), and the amide A band near 3100–3500 cm^−1^ (NH stretch) [39]. Then, a fluorescence spectrometer was used to characterize whether the gold nanocluster (GNC) colloid solution emitted electromagnetic waves. Emission of near-IR electromagnetic radiation from GNCs between 700 and 750 nm was observed under excitation at 350 nm, as shown in Figure 2c. 

To confirm that GNC-PLGA acts as a lipophilic fluorescent substance, ^1^H-NMR spectra verified the GNC-PLGA structure (Figure S5 in the Supplementary Materials) but showed peaks representing only the PLGA copolymer. Therefore, the chemical structure of GNC-PLGA was also further confirmed using FTIR spectroscopy (Figure 2b). Strong absorption from the molecular backbone of PLGA was observed, i.e., peaks at 1044–1176 cm^−1^ and 1716 cm^−1^ were attributed to the C-O-C stretching and symmetrical stretching of the ester bond (C=O), respectively. In addition, the alkyl bending vibration was observed at approximately 1542 cm^−1^. Notably, the amide A band near 3100–3500 cm^−1^ (NH stretch) on the surface of GNC disappeared completely after coupling with the PLGA molecule. This result was attributable to amide bond formation between the carboxyl group of PLGA and the amine of the GNC surface. Furthermore, the GNC-PLGA solution emitted near-IR electromagnetic radiation between 700 and 750 nm (Figure 2c). In particular, this near-IR waveband region not only reduced the background noise from the biomatrix but also actively enabled the imaging and tracking of cell fluorescence, meeting the experimental needs of cellular observation and image tracking in vivo. Therefore, the ^1^H-NMR spectra, FTIR spectra, and fluorescence spectrum confirmed the successful conjugation between GNCs and the PLGA chain.

### 2.3. Characterization of the Fluorescent NPs

The encapsulation of fluorescent substances loaded in APP NPs and PP NPs by precipitation-solvent evaporation was previously described. First, the particle size distribution results of blank PP NPs, GNC-PLGA/PP NPs, blank APP NPs, and GNC-PLGA/APP NPs were evaluated using dynamic light scattering (DLS) and are presented in Table 1. The average size of 213.5 nm for the _d_Asp_3_-PEG-PLGA copolymers to form blank APP NPs was greater than the average size of 130.5 nm for the PEG-PLGA copolymers to form blank PP NPs. Furthermore, the polydispersity index (PDI) value of blank APP NPs was also larger than that of blank PP NPs. Larger PDI values correspond to a larger size distribution in the APP NP sample, allowing the use of the polydispersity index (PDI) instead of the standard deviation [40]. This is because the bone-targeting moiety of the dendritic oligopeptide by three aspartic acids (_d_Asp_3_) caused the particles to become larger due to their hydrophilicity and thus increased the molecular weight of the copolymer.

**Figure 3:**
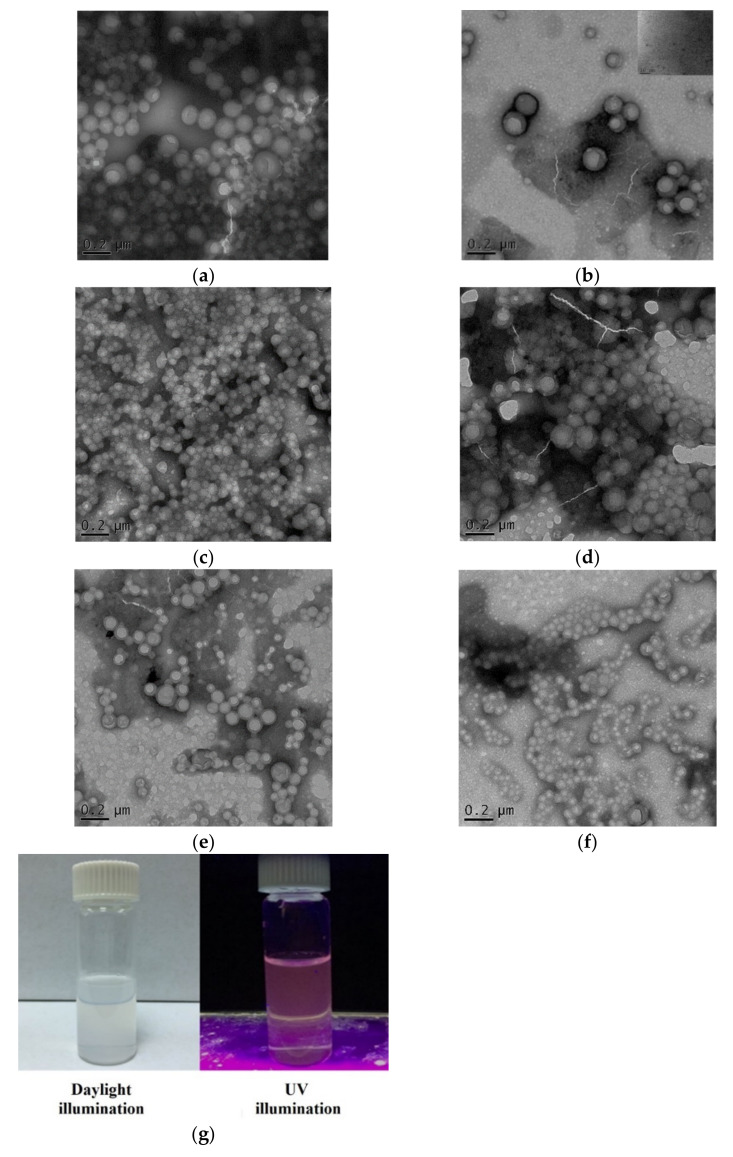
TEM images of _d_Asp_3_-PEG-PLGA (APP) NPs (**a**), GNC-PLGA/_d_Asp_3_-PEG-PLGA (GNC-PLGA/APP) NPs (**b**), PLGA-PEG-OMe (PP) NPs (**c**), GNC-PLGA/PLGA-PEG-OMe (GNC-PLGA/PP) NPs (**d**), simvastatin/_d_Asp_3_-PEG-PLGA (SIM/APP) NPs (**e**), and simvastatin/PLGA-PEG-OMe (SIM/PP) NPs (**f**), prepared using the solvent displacement method. Photos of the fluorescence emission of GNC-PLGA/APP NPs (weight ratio ~1/2) were observed under ultraviolet light (UV) and daylight illumination (**g**).

When the two copolymers _d_Asp_3_-PEG-PLGA and PEG-PLGA were further encapsulated with the fluorescent substance GNC-PLGA, the average particle diameters of both nanoparticles were slightly larger, at 247.8 nm and 225.5 nm, respectively. In addition, the polydispersity index (PDI) value of GNC-PLGA/APP NPs was slightly higher than that of GNC-PLGA/PP NPs. This phenomenon showed that the lipophilic substance of GNC-PLGA can increase the core volume of the hydrophobic area of nanoparticles, and this result is consistent with other literature reports on amphiphilic polymer-encapsulated hydrophobic substrates [20,30]. The spherical morphology and sizes of the above four nanoparticle systems (PP NPs, GNC-PLGA/PP NPs, APP NPs, and GNC-PLGA/APP NPs) were further confirmed by TEM observation, and the results are shown in Figure 3a–d. The presence of GNCs can also be observed in the TEM images, such as the GNC-PLGA/APP NPs shown in Figure 3d. In addition, the fluorescence emission photographs of GNC-PLGA/APP NPs in Figure 3g were observed clearly under ultraviolet light (UV) and daylight lamp illumination. Therefore, these results indicate that APP NPs and PP NPs can effectively encapsulate GNC-PLGA, and the encapsulation of GNC-PLGA/APP NPs and GNC-PLGA/PP NPs can be used to evaluate the endocytosis pathway and in vivo biodistribution.

### 2.4. Characterization of the SIM-Loaded APP NPs and PP NPs

To effectively transport SIM nanocarriers to the target bone tissue, SIM/APP NPs and SIM PP NPs must have the ability to remain in the bloodstream for a considerable amount of time. To avoid reticuloendothelial system (RES) uptake, the two most often-cited measures are the formation of a hydrophilic surface using PEG and the use of the optimal particle size [41,42]. For example, nanoparticles with diameters ≤ 5 nm rapidly undergo renal clearance upon intravenous administration [43]. Noncontinuous endothelia with vascular fenestrations measuring 50–100 nm are present in the liver, leading to nonspecific accumulation of larger particles [44]. Moreover, splenic filtration accounts for the retention of particles >200 nm due to the 200–500 nm size range of interendothelial cell slits [45].

The particle size distribution and related morphology of SIM-loaded APP (SIM/APP) NPs and SIM-loaded PP (SIM/PP) NPs were also observed through dynamic light scattering (DLS) and transmission electron microscopy (TEM) images. The sizes of SIM/APP NPs and SIM/PP NPs were ~200 nm and ~100 nm, respectively. Both SIM-loaded NPs were smaller than the blank APP NPs and blank PP NPs, as shown in Table 1 and Table 2. The reason for this finding may be that the amount of SIM loading was still small, and SIM is also a lipophilic molecule that can tightly arrange the core molecules of PLGA. The polydispersity index (PDI) values of SIM/APP NPs and SIM/PP NPs are approximately 0.474 and 0.121, respectively, similar to those of previous GNC-PLGA/APP NPs and GNC-PLGA/PP NPs. Figure 3e,f shows the morphologies of SIM/APP NPs and SIM/PP NPs determined through TEM, from which we can see that both NPs were approximately spherical. Thus, their particle sizes and distributions were consistent with the results of DLS and PDI. In addition, the zeta potentials of SIM/APP NPs and SIM/PP NPs were approximately −35.5 mV and −25.5 mV, respectively. The reason for these results is that the bone-targeting moiety _d_Asp_3_ caused a more negative charge of the electrical double layer of nanoparticles than the OMe group of PP NPs. However, nanoparticles with neutral (1.3 mV) or negative (−10.6 mV) surface charges have been shown to reduce the adsorption of serum proteins, resulting in longer circulation half-lives and lower accumulation in the liver and spleen [46,47]. Positively charged nanoparticles have a higher rate of nonspecific uptake in most cells [42]. Alternatively, the system size and negative charge of the pegylated drug nanocarrier should be 100–200 nm and <−10 mV, respectively, and the NPs are also expected to reach the bone tissue via distribution in the blood vessels and living organs of interest. The average size of both negatively charged NPs carrying SIM was more than 100 nm but less than 200 nm. Therefore, both SIM NPs are expected to be taken up by bone marrow-derived mesenchymal stem cells (BMSCs), and they can also enter the circulatory system in vivo. The DL% and EE% are shown in Table 2. The drug encapsulation efficiencies of SIM/APP NPs and SIM/PP NPs were 72.48% and 76.29%, respectively, and the drug loadings were 0.28 μg/mg and 0.31 μg/mg, respectively. These results indicate that SIM/APP NPs and SIM/PP NPs can effectively encapsulate SIM.

### 2.5. Evaluation of the Cellular Uptake Pathway

Previous studies reported that NPs were internalized through endocytosis pathways, including clathrin-mediated, caveolae-mediated endocytosis, and micropinocytosis pathways [48,49]. Herein, inhibitors of these pathways were used to investigate the mechanism of cellular uptake of GNC-PLGA/APP NPs on D1 cells. First, flow cytometry is a fluorescence-based method in which a suspension of cells is passed across one or several lasers in such a way that only one cell faces the laser at a defined time to allow quantification per cell. However, the near-IR cell fluorescence intensity distributions on a logarithmic scale were used to evaluate the endocytosis pathway because of the GNC-PLGA/APP NP system. In these cases, there is evidence that cells within the population have internalized varying amounts of GNC-PLGA/APP NPs (note the logarithmic scale in Figure S6a–e in the Supplementary Materials. The untreated cells had a low background fluorescence signal, while the cells exposed to 30 μL of GNC-PLGA/APP NPs and different endocytosis inhibitors—such as a blank control with no inhibitor, hypertonic sucrose as a clathrin-mediated endocytosis inhibitor, rottlerin as a micropinocytosis inhibitor, and MβCD as a caveolae-mediated endocytosis inhibitor—exhibited different fluorescence values. The near-IR signal of GNC-PLGA/APP NPs without an inhibitor (Figure S6b) exhibited much higher fluorescence intensity due to particle uptake. One of the inhibitors was hypertonic sucrose, which inhibits clathrin-mediated endocytosis and has a strong ability to inhibit the endocytosis of GNC-PLGA/APP NPs by D1 cells; the results are shown in Figure S6c, demonstrating the involvement of the clathrin-mediated pathway. In addition, inhibitors of micropinocytosis and caveolae-mediated endocytosis exhibited much higher fluorescence intensity due to particle uptake, as shown in Figure S6d,e.

**Figure 4:**
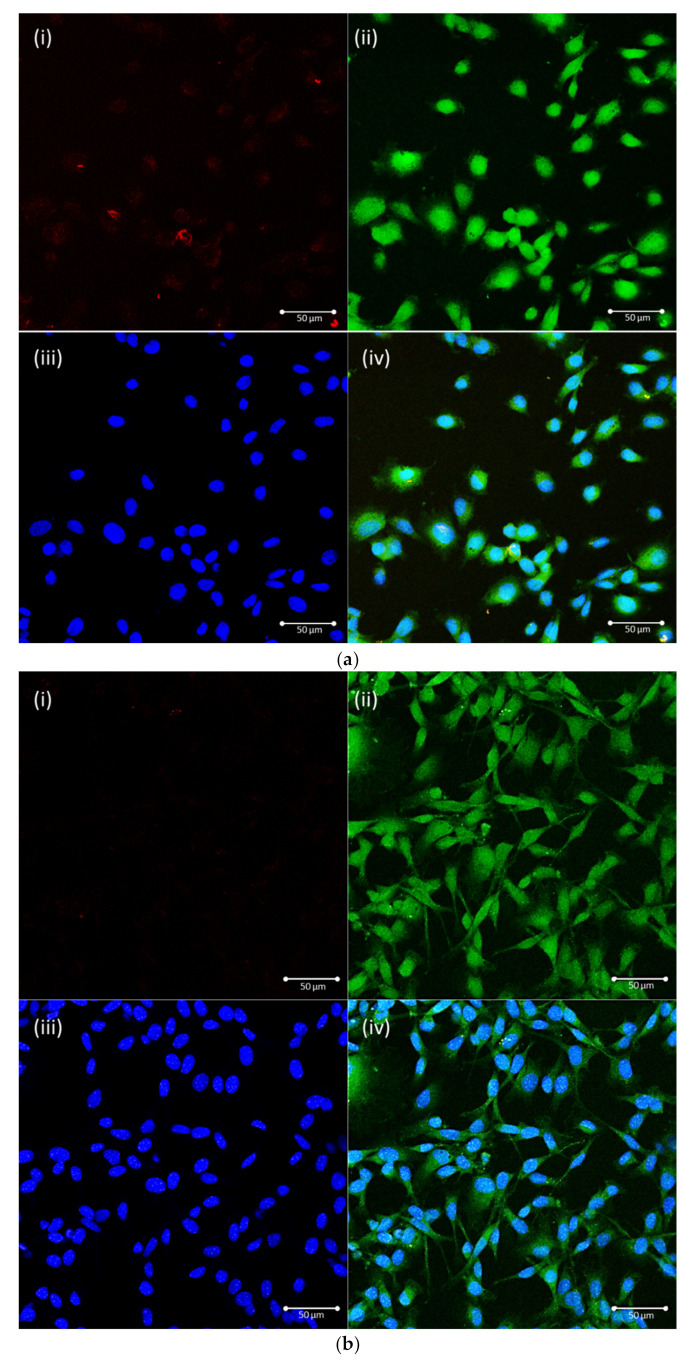
Cellular uptake evaluated for GNC-PLGA/APP NPs in D1 cells. Culture conditions without inhibitor (**a**) and with the inhibitor of hypertonic sucrose solution (**b**). Cellular uptake in D1 cells (a bone marrow-derived mesenchymal stem cell line) was incubated with GNC-PLGA/APP NPs for 10 min. Red: GNC-PLGA/APP NPs (**i**); green: FITC-labeled cell cytoplasm (**ii**); blue: DAPI-stained cell nucleus (**iii**). Overlapping images (**iv**) from (**i**), (**ii**), and (**iii**). Scale bar = 50 μm.

To further comprehend the intracellular pathway involved in clathrin-mediated endocytosis, the colocalization of NPs was investigated via fluorescence microscopy. FITC was used to label the cell cytoplasm green, and DAPI was used to stain the cell nucleus blue in this study. D1 cells were preincubated for 30 min with 200 mM hypertonic sucrose solution as an endocytic inhibitor, and GNC-PLGA/APP NPs were added and incubated for 10 min at 37 °C. The other group had no added inhibitor. The results presented in Figure 4a show that the red fluorescence signal was clearly observed in the no-inhibitor group, but the red fluorescence signal was almost absent in the hypertonic sucrose group in Figure 4b. These results show that APP NPs had effective cell uptake behavior through clathrin-dependent endocytosis, mainly based on dendritic (aspartic acid)_3_-functionalized PEG as a bone-targeting ligand. Therefore, APP NPs are expected to effectively deliver SIM to cells to achieve the desired endocytosis function.

### 2.6. Effects of the Formation of Mineralized Nodules

Our previous study demonstrated that 0.5 μM SIM was an effective dose for inducing BMSC osteogenesis of D1 cells without causing cytotoxicity, as shown using the alizarin red staining method [36]. To evaluate the effects of SIM/APP and SIM/PP NPs, which represent a 0.5 μM SIM concentration based on a theoretical calculation, on the osteogenic differentiation of D1 cells, we also used alizarin red staining to evaluate the matrix mineralization. A 0.5 μM SIM group was used as the positive control, and the control group was not treated with SIM. An osteogenic medium was used to induce bone differentiation in each group.

**Figure 5:**
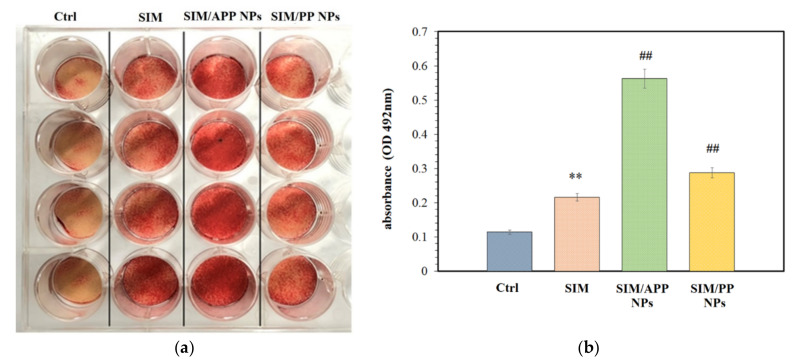
Effects of SIM/APP NPs, SIM/PP NPs, and positive control consisting of free simvastatin on mineralization in D1 cells. Optical photographs of various groups of wells stained with alizarin red S (**a**) and their ELISA quantitative analysis results (**b**). Values are the mean ± SD, *n* = 3. (**) indicates significant differences of *p* < 0.005 compared with the control group (Ctrl), (##) indicates significant differences of *p* < 0.005 compared with the SIM group.

Figure 5a shows images of alizarin red-stained cells from each treated group and the control group cultured in an osteogenic medium. More mineralized nodules formed when SIM was added to the medium. The quantitative analysis also showed a significant difference compared with the control group (Figure 5b). In particular, the cultures treated with 0.5 μM SIM/APP NPs showed significantly higher degrees of D1 mineralization than those treated with 0.5 μM SIM and SIM/PP NPs. However, the differentiation of D1 cells into the osteoblastic lineage is a complex process that includes D1 cell adhesion, proliferation, differentiation, maturation, and mineralization [50]. Considering the inductive effect of aspartic acid on osteogenic differentiation, this might be one of the reasons for the greater expression of mineralized nodules upon treatment with SIM/APP NPs than upon treatment with SIM and SIM/PP NPs, which could be related to the presence and more influential role of aspartic acid in the integrin-binding motif [51]. Deshpande et al. also reported that poly-Asp sequences act as analogs of noncollagenous acidic proteins when deposited on purified collagen nanofibers, resulting in significantly improved mineralization [52]. The detailed mechanism of SIM/APP NP-triggered D1 cell mineralization remains unclear.

### 2.7. In Vivo Distribution Assay in Rats

**Figure 6:**
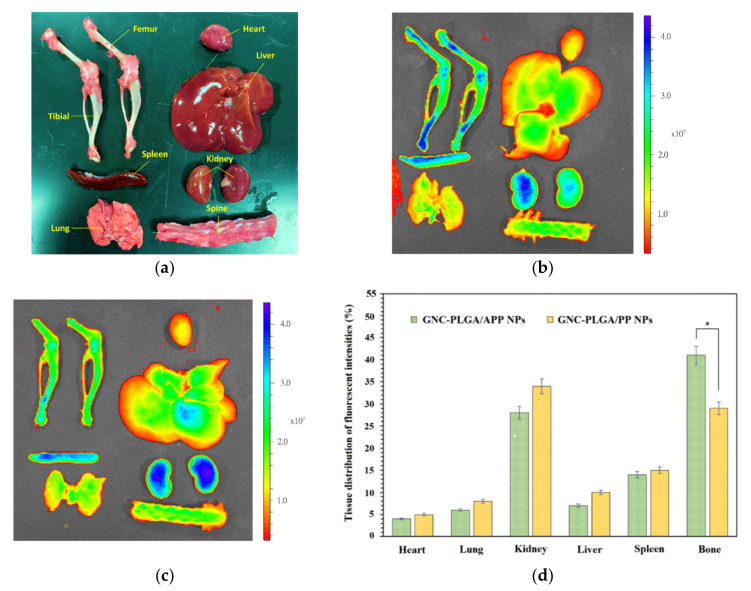
The distribution and fluorescence quantification in vivo were evaluated after 6 h of injection of both nanoparticle groups (GNC-PLGA/APP NPs and GNC-PLGA/PP NPs) into the tail vein of SD rats. Optical photographs of various major tissues (heart, liver, spleen, lung, kidney) and bone (bilateral femur/tibia, and spine) of SD rats (**a**) and fluorescence images of GNC-PLGA/APP NPs (**b**) and GNC-PLGA/PP NPs (**c**) were taken for the major tissues. Fluorescence quantitative results of the major tissues are shown in (**d**). These results represent the means ± SDs (*n* = 3). (*) indicate significant differences of *p* < 0.05).

In vivo tests evaluating the whole-body distribution of NPs indirectly assess the abilities of bone-targeted APP NP delivery systems. The fluorescent intensities in Figure 6a–c were evaluated through GNC-PLGA/APP NPs and GNC-PLGA/PP NPs, which accumulated in the skeleton 6 h after intravenous administration via the tail vein in rats. As shown in Figure 6d, the fluorescence amounts of both NPs were detected mainly in kidney and bone tissues (note: bone including bilateral femur/tibia and vertebrae) rather than in the heart, liver, spleen, and kidney. The accumulation of fluorescent signals of GNC-PLGA/APP NPs and GNC-PLGA/PP NPs in the kidney is explained by the fact that the metabolism of the hydrophilic GNCs is dominated by the renal pathway [43,53]. This result is very different from that obtained previously with the IR-780 fluorescent agent, which accumulates in the lung [35]. This is because IR-780 is a lipophilic fluorescent agent, and it will be metabolized through the liver. However, the lipophilic simvastatin of SIM/APP NPs, after entering the systemic circulation, is distributed in the whole body, absorbed by other tissues, metabolized by the liver, and removed by the kidneys [35]. This is the reason why the excessive intake of statins causes more concern about liver toxicity than kidney toxicity. In addition, Figure 6d shows the accumulation of both NPs in the skeleton (represented by the bilateral femur/tibia and vertebrae), and the fluorescence intensities in the bone from the GNC-PLGA/APP NPs were clearly higher than those from the GNC-PLGA/PP NPs. Fluorescence quantification clearly shows that 41% of GNC-PLGA/APP NPs accumulate in bone tissues, which is higher than the 29% of GNC-PLGA/PP NPs. Thus, the former is expected to be beneficial for achieving more effective bone formation and reducing liver toxicity or the possibility of rhabdomyolysis.

### 2.8. In Vivo Experiments with OPs Models in Rats

Systemic administration, either orally or via intravenous (IV), intramuscular (IM), intraperitoneal (IP), or subcutaneous (SC) injections, is a simple and safe method to deliver SIM. However, in this approach, the entire body is affected, most of the drug is metabolized through the liver, and its concentration and bioavailability decrease quickly and time-dependently [23,54]. Thus, only a small concentration of the administered SIM may reach the target bone tissue through the general formulation of SIM. Moreover, SIM has a low affinity for bone. According to A. Oryan et al. [23], the relevant literature reported that statins have been systemically administered in vivo at different doses ranging from 0.1 to 120 mg/kg. The most effective doses are between 10 and 120 mg/kg in vivo. Therefore, it is necessary to maintain the effective concentrations of SIM at the bone loss site for a long time to ensure that SIM NPs (or free SIM) can continuously affect OP via bone targeting. Some in vivo investigations used systemic delivery of SIM and SIM NPs in experimental models relevant to OP and important results are shown in Table 3 [29,30,55,56,57]. A dosage of 0.5 to 1.0 mg of SIM/kg of bone-targeting SIM NPs by intravascular (IV) injection given every 2 days was effective for bone formation in a postmenopausal (OVX) OP rat model.

Based on our previous in vivo distribution assay results, we suggested once again that the bone-targeting moiety of dendritic oligopeptides formed by three aspartic acid molecules (_d_Asp_3_) of APP NPs could target bone, and the expected reduction in side effects in other organs was affected by SIM. Moreover, we compared related low-dose SIM treatment (0.25 mg/kg per time, and 2 times per week) for both OP rat models (the primary type, OP model with OVX animals, and the secondary type, OP model with disuse animals) to prove the therapeutic effect of this bone-targeting APP NPs system compared to the non-bone-targeting system of PP NPs.

**Figure 7:**
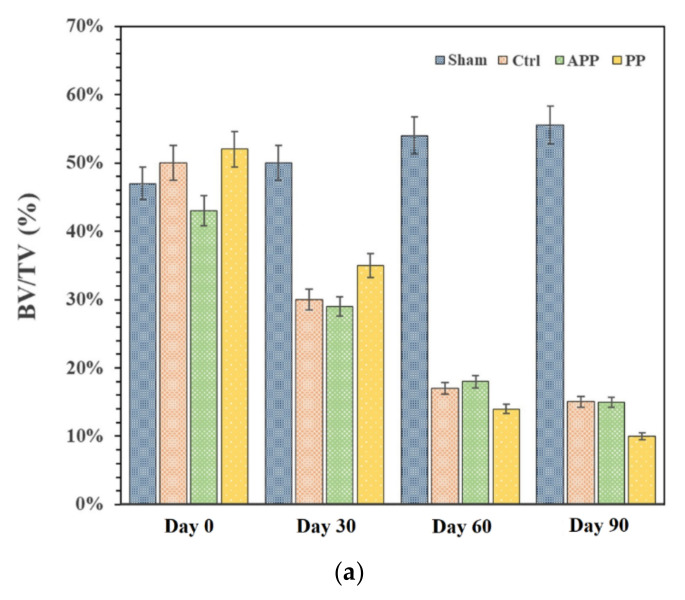

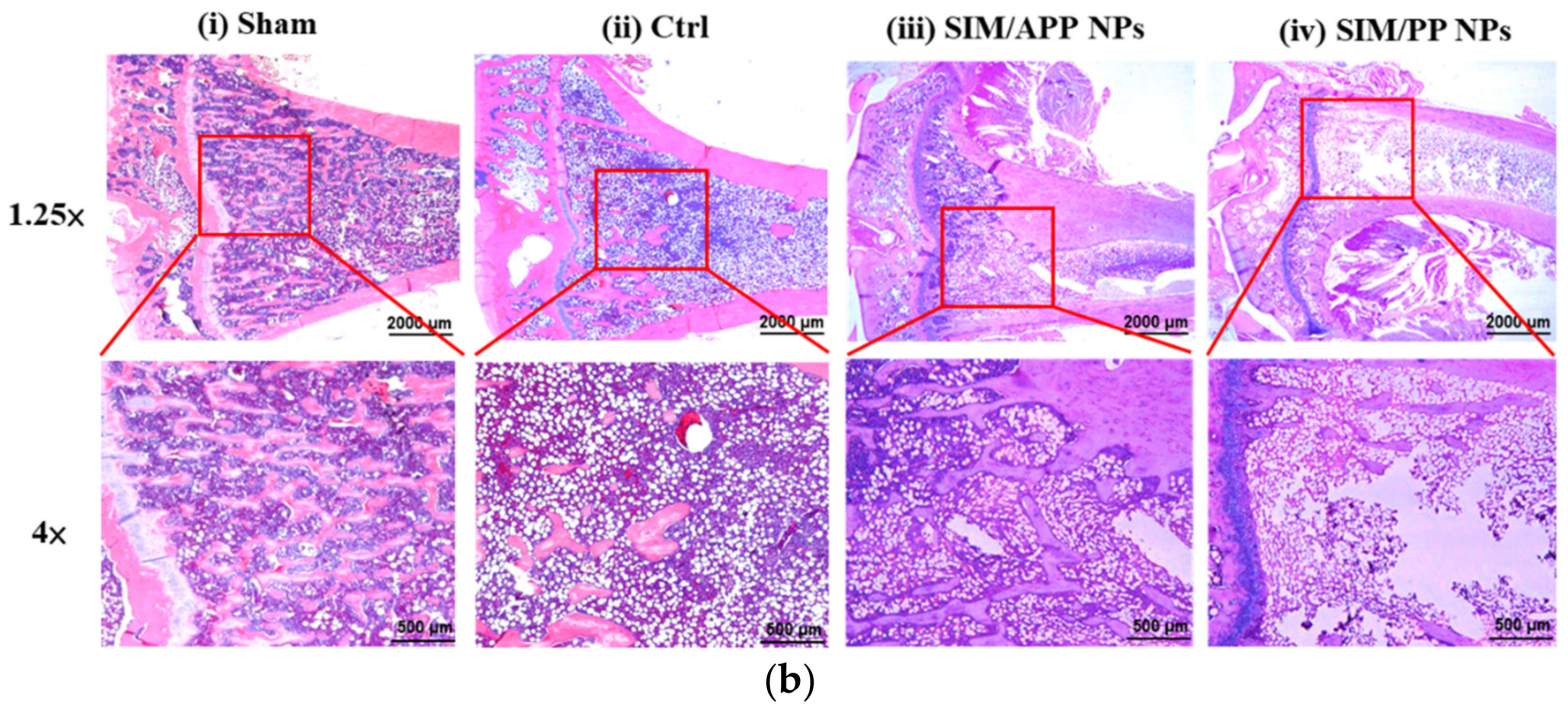
The bone formation effectiveness of SIM nanocarriers in postmenopausal osteoporosis SD rat models after OVX for 3 months. There were the following four groups: (**i**) sham group: no OVX surgery and no SIM drug treatment; (**ii**) control (Ctrl) group: OVX surgery and no SIM treatment; (**iii**) SIM/APP NP group: OVX surgery and SIM/APP NP treatment; and (**iv**) SIM/PP NP group: OVX surgery and SIM/PP NP treatment. Micro-CT was used to analyze the distal portion of the tibia bone, and the bone volume to total volume (BV/TV) ratio results represent the means ± standard deviation (*n* = 3) (**a**). H&E staining also revealed the distal portion of the tibia bone tissue histologic sections (**b**).

The bone volume-to-total volume (BV/TV) ratio can be extensively used as a predictor for evaluating OP [13]. First, micro-CT scanning was used to analyze the trabecular bone volume percentage (BV/TV) of the distal portion of the tibia bone at 12 weeks post-ovariectomization, as shown in Figure 7a, and the related quantification method via grayscale CT Analyzer (Skyscan 1076, Belgium Bruker, Billerica, MA, USA) software is shown in Figure S7 in the Supplementary Materials. The BV/TV values were distinctly different between the sham group and the positive OVX group, with a value of 55.8% in the sham group and 15.3% in the OVX group. The BMD of the OVX group was reduced by 72.58% compared to that of the sham group, indicating that the osteoporotic model rats were established successfully. In addition, it was not clearly observed that the BV/TV values of all treatment groups were significantly increased compared to those of the OVX group after 12 weeks, given the BV/TV values of 15.1% in the OVX with SIM/APP NPs group and 10.6% in the OVX with SIM/PP NPs group. Further histochemical staining analyses of the OVX model are shown in Figure 7b, which reveal similar micro-CT situations of the four groups (sham, Ctrl, SIM/APP, SIM/PP). Notably, the OVX group (Ctrl) showed sparse loss of interconnectivity and thinning of the trabeculae, thereby showing widened intertrabecular spaces. Furthermore, the OVX group showed more adipose tissue than the other groups. However, there was no statistically significant increase in the BV/TV ratio of the SIM/APP NP-treated groups in comparison to the OVX group. It appears that the interconnectivity of the trabeculae in this phenomenon was slightly better than that of the OVX group. As reported in the literature [10], both bone resorption and bone formation are increased in postmenopausal osteoporosis, but the extent of increased bone resorption exceeds that of augmented bone formation, which still causes OP. The main possible reason explaining why, in this case, even with bone-targeting ability, SIM/APP NPs still cause poor OP treatment results after OVX is that the postmenopausal OP model involves systemic bone loss [7,58], and the therapeutic concentration of bone-targeting SIM/APP NPs to reach whole-body bone loss under systemic circulation is still too low. In particular, compared with other studies, our study found that the SIM concentration was at least half as low. Unfortunately, there is no single all-encompassing treatment for all types of OP. Therefore, OVX causes a higher severity of whole-body bone loss and may require more effective bone formation doses for SIM/APP NPs in the future.

**Figure 8:**
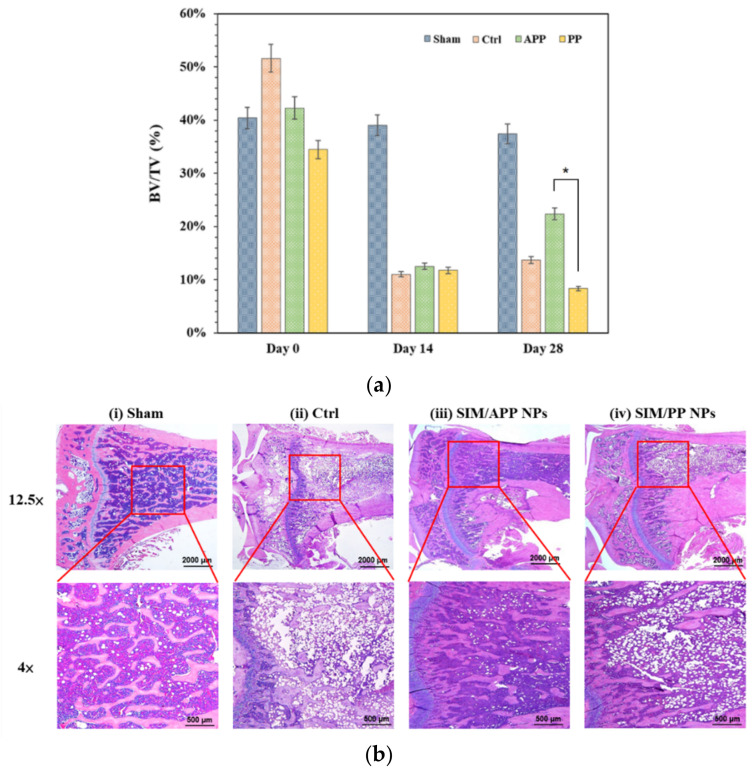
The effective bone formation ability of SIM nanocarriers in the disuse osteoporosis SD rat model after hindlimb suspension for 1 month. There were the following four groups: (i) sham group—no hindlimb suspension and no SIM drug treatment; (ii) control (Ctrl)—hindlimb suspension and no SIM treatment; (iii) SIM/APP NP group—hindlimb suspension and SIM/APP NP treatment; and (iv) SIM/PP NP group—hindlimb suspension and SIM/PP NP treatment. Micro-CT was used to analyze the distal portion of the tibia bone, and the bone volume to total volume (BV/TV) ratio results represent the means ± standard deviation (*n* = 3) (**a**). (*) indicates significant differences of *p* < 0.05. H&E staining also revealed the distal portion of the tibia bone tissue histologic sections (**b**).

On the other hand, the disuse OP model focuses on the immobilization-induced and local loss of bone density based on the inhibition of osteoblast-mediated bone formation and the acceleration of osteoclast-mediated bone resorption [58,59], and it has been demonstrated that SIM/APP NPs injected at the same lower dose (0.25 mg/kg per time and 2 times per week for 3 weeks) result in a better tibia bone density, as shown in Figure 8. For example, micro-CT scanning was also used to analyze the trabecular bone volume percentage (BV/TV ratio) at 4 weeks after the hindlimb suspension model had been established for 1 month. The BV/TV ratios at the distal portion of the tibia bone were distinctly different between the sham group (no hindlimb suspension) and the control group (hindlimb suspension rats only), with a value of 37.8% in the sham group and 13.8% in the control group. The BMD of the control group was reduced by 63.49% compared to that in the sham group, indicating that the osteoporotic model rats were established successfully [60,61]. It can be clearly observed that the BV/TV ratio of the SIM/APP NP group was 22.1%, which was significantly higher than the BV/TV ratios of the control group and the SIM/PP NP group, with values of 8.1% and 13.8%, respectively, as shown in Figure 8a. Furthermore, the bone-targeting groups of SIM/APP NPs showed a significant increase in BMD and the BV/TV ratio when compared to the control group of hindlimb suspension rats only and the non-bone-targeting groups of the SIM/PP NP. In other words, the bone density recovery efficiency of the SIM/APP NP group was still higher than that of the control group by approximately 60%. Furthermore, the bone density recovery efficiency of the SIM/APP NP group was 172% higher than that of the SIM/PP NP group. The histochemical staining analyses shown in Figure 8b also revealed a significant difference between the SIM/APP NP groups and the control and SIM/PP NP groups. The SIM/APP NPs showed more complete interconnectivity of the bone trabeculae and thickening of the bone trabeculae, thereby showing narrow intertrabecular spaces and indicating a significant improvement in bone quality.

This finding is important because the modification of the dendritic oligopeptide moiety by three aspartic acid molecules (_d_Asp_3_) can increase the distribution of SIM/APP NPs in bone tissue, as shown by the in vivo distribution of GNC-PLGA/APP, thereby increasing the local concentration of SIM at the high-minerality bone site; significantly stimulating osteogenic gene expression of RUNX-2, BMP-2, alkaline phosphatase, and osteocalcin; and increasing the efficacy of bone formation. The dendritic oligopeptide of _d_Asp_3_ is negatively charged and able to bind free calcium ions near the surface of local severe OP in a disuse model. A comparison of the OP model with the related low-dose SIM treatment (0.25 mg/kg per dose and 2 times per week for 3 weeks) for the secondary-type model of disuse in animals proved the therapeutic effect of this bone-targeting APP NP system compared to the non-bone-targeting system of SIM/PP NPs. Furthermore, simvastatin is a lipophilic drug and could be used as an encapsulated lipophilic model drug and the potential research object of the bone-targeting nanocarrier. Meanwhile, the SIM/APP NP developed for this research will also have potential clinical applications in the future.

## 3. Materials and Methods

### 3.1. Materials

L-Aspartic acid (HO_2_CCH_2_(NH_2_)CO_2_H), (±)-α-lipoamide (C_8_H_15_NOS_2,_ ≥99%), gold(III) chloride trihydrate (HAuCl_4_·3H_2_O, 99%), sodium borohydride (NaBH_4_, 98%), *N*-(3-dimethylaminopropyl)-*N*’-ethylcarbodiimide hydrochloride, fluorescein isothiocyanate (C_21_H_11_NO_5_S, FITC), 2-(4-amidinophenyl)-1*H*-indole-6-carboxamidine (C_16_H_15_N_5_, DAPI), nuclear magnetic resonance (NMR) d-solvents, tetramethylsilane (TMS), calcium hydride, and PLGA (50/50, *M*_w_ 50,000–75,000, no. 430447) were purchased from Sigma-Aldrich Co. (Burlington, MA, USA). Boc-Asp-OH (tert-butyl carbamates-Asp-OH) was purchased from Fluka^®^ (Sigma-Aldrich Co., USA). H_2_N-PEG-COOH (*M*_w_ 3400) and H_2_N-PEG-OMe (*M*_w_ 3400) were purchased from Laysan Bio., Inc. (Arab, AL, USA). 3-(ethyliminomethyleneamino)-*N*,*N*-dimethyl-propan-1-amine (EDC), *N*,*N*’-dicyclohexylcarbodiimide (DCC) and *N*-hydroxysulfosuccinimide (Sulfo-NHS) were purchased from Acros Organics (Pittsburgh, PA, USA). Trifluoroacetic acid, hydrochloric acid, triethylamine (TEA), and *N*,*N*-dimethylformamide (DMF) were purchased from Riedel-de Haen^®^ (Sigma-Aldrich Co., Burlington, MA, USA). Sodium hydroxide and magnesium sulfate anhydrous powder were purchased from Showa PK Co. (Tokyo, Japan). SIM was purchased from Merck Inc. (Kenilworth, NJ, USA). All other solvents were purchased from TEDIA (Fairfield, OH, USA) or J. T. Baker (Phillipsburg, NJ, USA). The other chemicals were of analytical/reagent grade and were used without further purification.

### 3.2. Synthesis of _d_Asp_3_-PEG-PLGA and PEG-PLGA

The bone-targeting functional amphiphilic block copolymer of _d_Asp_3_-PEG-PLGA (APP) was synthesized by esterification and amide bond formation in our previous study, as shown in Figures S1–S3 in the Supplementary Materials [35]. First, the bone-targeting moiety of the dendritic oligopeptide composed of three aspartic acid molecules (_d_Asp_3_) was synthesized as follows: the two carboxyl groups of aspartic acid were methylated to form an Asp_1_-(OMe)_2_ moiety by esterification, 15 mg of aspartic acid was dissolved in 100 mL of MeOH, and then 16.4 mL of SOCl_2_ was slowly added in an ice bath. This solution was heated and refluxed for 5 h and then dried using a vacuum system to create powder Asp_1_-(OMe)_2_. Boc-Asp-OH (1.475 g), Asp_1_-(OMe)_2_ (2.5 g), and DCC (3.2 g) were dissolved in 25 mL of CHCl_3_ to form dendritic Boc-_d_Asp_3_-(OMe)_4_ by amide bond formation, and then 1.77 mL of TEA was added to react for 24 h at room temperature. A glassy filter was used to remove the precipitate, which was then dried using a vacuum system to produce a pale yellow powder dendritic _d_Asp_3_-(OMe)_4_.

Similarly, during the formation reaction, the product NH_2_-PEG-_d_Asp_3_ was synthesized by dendritic _d_Asp_3_-(OMe)_4_ and NH_2_-PEG-COOH via amide bond formation. Then, 100 mg of NH_2_-PEG-COOH, 36.98 mg of dendritic _d_Asp_3_-(OMe)_4_, 8.45 mg of EDC, and 5.07 mg of sulfo-NHS were dissolved in 5 mL of DMF, and 0.1 mL of TEA was added to react for 24 h at room temperature. Then, the mixture was dialyzed against deionized water using a dialysis membrane (*M*_w_ 3.5 kDa) for 48 h, and the pale yellow product was freeze-dried to generate NH_2_-PEG-_d_Asp_3_-(OMe)_4_. This NH_2_-PEG-_d_Asp_3_-(OMe)_4_ was dissolved in a few MeOH solutions and reacted with 0.5 M NaOH to remove the methoxy group (OMe) at pH 12 for 4 h. Then, 1 M HCl was added to the solution to return to pH 7, and the mixture was dialyzed against deionized water using a dialysis membrane (*M*_w_ 3.5 kDa) for 48 h. The pale yellow product was freeze-dried to generate NH_2_-PEG-_d_Asp_3_.

The final bone-targeting functional amphiphilic block copolymer of _d_Asp_3_-PEG-PLGA (APP) was also synthesized by an amide bond reaction between PLGA and NH_2_-PEG-_d_Asp_3_. Three hundred milligrams of PLGA, 100 mg of NH_2_-PEG-_d_Asp_3_, and 8 mg of DCC were dissolved in 5 mL of DMF, and then 0.1 mL of TEA was added to react for 24 h at room temperature. The mixture was dialyzed against DMF using a dialysis membrane (*M*_w_ 12 kDa) for 48 h and then dialyzed against deionized water for 48 h. Then, the solution was freeze-dried to obtain _d_Asp_3_-PEG-PLGA [35]. The synthesized PEG-PLGA (PP) amphiphilic copolymer was used as a control group and synthesized as described previously [35]. In brief, 300 mg of PLGA, 100 mg of NH_2_-PEG-OMe, and 8 mg of DCC were dissolved in 5 mL of DMF, and then 0.1 mL of TEA was added to react for 24 h at room temperature. The mixture was dialyzed against DMF using a dialysis membrane (*M*_w_ 12 kDa) for 48 h and then dialyzed against deionized water for 48 h. Finally, the solution was freeze-dried to obtain the block copolymer of PEG-PLGA (PP).

### 3.3. Preparation of GNCs, Florescence NPs, and SIM NPs

We used the bone-targeting functional amphiphilic block copolymer_d_Asp_3_-PEG-PLGA (APP) to precipitate APP nanoparticles (NPs). However, the biodistribution data of bone-targeting APP NPs carrying an IR-780 fluorescent reagent in Sprague–Dawley (SD) rats showed that the average fluorescence intensity from the liver and lung was still significantly higher than that in a previous study [35]. A possible reason for this finding is that the fluorescent reagent IR-780 is metabolized through the liver and trapped in the capillary bed of the normal vasculature of the lung [35]. To further re-evaluate the in vivo biodistribution of APP bone-targeting NPs in SD rats, we replaced the fluorescent reagent IR-780 with biocompatible gold nanoclusters (GNCs) with near-IR fluorescence. In addition, we used GNC fluorescence tracing to understand the endocytosis pathway of bone marrow-derived mesenchymal stem cells (BMSCs) for APP NPs under in vitro cell culture conditions.

GNCs were prepared in the same way as reported in an earlier study but with some modifications [62]. Here, 0.036 g of lipoamide was dissolved in 2.5 mL of DMF, and then 3 mL of NaOH aqueous solution (0.5%) was added for a ring-opening reaction. Next, 6 mL of HAuCl_4_ solution (0.01 M) and 6 mL of NaBH_4_ solution (0.02 M) were sequentially added and stirred in an ice bath for 30 min. The resultant GNCs were isolated by methanol-induced agglomeration. The solution was vacuum-dried and redissolved in DMF, resulting in the addition of an amino group (-NH_2_) as a functional group on the GNC surface; the scheme is shown in Figure S4 in the Supplementary Materials.

The purpose of coupling GNC with PLGA to form GNC-PLGA is to generate a fluorescent substance that can be carried within the interior of APP NPs due to its hydrophobicity; this coupling was accomplished by combining PLGA and GNC via an amide bond reaction. To prepare the activation intermediate at the corresponding carboxyl groups (-COOH) on PLGA, 100 mg of PLGA (50/50) was activated with 3 mg of DCC in 1 mL of anhydrous DMF, and then 1 mL of GNC solution was added to form GNC-PLGA via amide bonds. The reaction mixture was stirred overnight at room temperature. The solution was dialyzed (MWCO 12,000 Da) against DMF for 12 h to remove the unreacted GNCs and DCC. The resulting solution was further dialyzed against distilled water for 3 days to remove the DMF and then freeze-dried. A schematic of the GNC-PLGA synthesis is shown in Figure S8a in the Supplementary Materials, and chemical confirmation was obtained by proton nuclear magnetic resonance spectroscopy (NMR; Varian Gemini-200, Palo Alto, CA, USA) and Fourier transform infrared spectroscopy (FTIR; System 2000 FT-IR, Perkin Elmer, Waltham, MA, USA). GNC-PLGA was dissolved in a deuterated chloroform solvent, and ^1^H NMR spectra were recorded on an NMR spectrometer. FTIR spectra of GNC and GNC-PLGA were acquired to confirm the chemical structures in the spectral range of 400–4000 cm^−1^ at a resolution of 4 cm^−1^.

The fluorescent NP system was fabricated and included the bone-targeting group GNC-PLGA/APP NPs (weight ratio ~1/2) and the non-bone-targeting control group GNC-PLGA/PP NPs (weight ratio ~1/2). Both GNC-PLGA/APP NPs and GNC-PLGA/PP NPs were also prepared using a similar solvent displacement method [35,63]. Briefly, 20 mg of APP or PP and 10 mg of GNC-PLGA were dissolved in 800 µL of acetone, and then 2500 µL of deionized water was added. A diagram of the formation is shown in Figure S8b. Finally, the solvent was removed under reduced pressure.

To evaluate the mineralization ability of APP NPs and PP NPs in vitro and their bone-targeting therapy in vivo, both NP systems carried SIM. The SIM/APP NP and SIM/PP NP drug nanocarriers were prepared using a previously described solvent displacement method. Briefly, 20 mg of APP or PP and 1 mg of SIM were dissolved in 800 µL of acetone, followed by the addition of 2500 µL of deionized water, and the solvent was removed under reduced pressure.

All of the above nanoparticle systems comprising GNCs, GNC-PLGA/APP NPs, GNC-PLGA/PP NPs, SIM/APP NPs, and SIM/PP NPs were characterized in terms of morphology and size range by transmission electron microscopy (TEM; JEOL JEM-2100, Akishima, Tokyo, Japan) under phosphotungstic acid (1%) staining. The surface functional groups of GNCs were checked by Fourier transform infrared spectroscopy (FTIR; System 2000 FT-IR, Perkin Elmer, USA). To confirm the fluorescence emission of the GNC NPs, GNC-PLGA, GNC-PLGA/PP NPs, and GNC-PLGA/APP NPs before in vitro or in vivo studies, the initial fluorescence emission intensities were measured using a fluorescence spectrometer (Varian, Cary Eclipse) with emission at 350 nm. In addition, these nanoparticle systems were analyzed for their zeta potential and particle size distributions using a zeta potential instrument (Zetasizer 3000-HSA and Mastersizer M-2000 P-III, Malvern).

### 3.4. Cell Culture and Related Evaluations

Mesenchymal cells (D1, American Type Culture Collection, Manassas, VA, USA), cloned from BALB/c mouse bone marrow cells, were cultured according to the supplier’s (Invitrogen, Carlsbad, CA, USA) recommendations in bone medium (Dulbecco’s modified Eagle medium; DMEM). The DMEM contained 10% fetal bovine serum and 0.1% sodium ascorbate in a humidified atmosphere of 5% CO_2_ at 37 °C.

#### 3.4.1. Cellular Uptake Pathway Study

For pharmacological inhibition of clathrin-mediated endocytosis (200 mM hypertonic sucrose) [48,49,64], caveolae-mediated endocytosis (10 mM methyl-beta-cyclodextrin; MβCD) [48,49], and micropinocytosis (2 μM rottlerin) [48,49], D1 cells were preincubated with the treatment solutions, and fresh medium containing 30 μL of GNC-PLGA/APP NPs was added. In brief, 5 × 10^5^ D1 cells were preincubated for 30 min with inhibitor solution in 6-well plates. After preincubation, the medium was replaced with 1 mL of GNC-PLGA/APP NPs in DMEM, and the cells were incubated for 10 min. Then, the cells were trypsinized and washed thoroughly with PBS twice by centrifugation, and the supernatant was removed and redissolved in 1 mL of PBS. The relative fluorescence intensity was measured using a flow cytometer (FACS Vantage-SE, Becton Dickinson), and untreated cells were used as a control. A typical forward scattering–side scattering (FS-SS) double scatter plot together with the fluorescence intensity distribution of the cells exposed to fluorescently labeled nanoparticles and healthy cells were easily distinguished from that of the cell debris, which typically has much lower linear FS and SS signals. The signals were transformed into side scattering intensity (SSC) histogram plots to visualize the comparisons among detection channels. Based on the above possible uptake pathway analysis, APP nanoparticles were taken up through inhibitor-dependent endocytosis. 

Further re-evaluation of the uptake pathway of D1 cells by fluorescence microscopy observation was performed via specific inhibitor-dependent endocytic inhibition experiments. D1 cells were incubated with media containing fluorescein isothiocyanate (FITC) and then preincubated for 30 min with an endocytic inhibitor solution. After preincubation, GNC-PLGA/APP NP colloids were added to cell cultures and incubated for 10 min at 37 °C. At the end of the incubation, the cells were fixed with 10% formalin–saline for 10 min and then washed thoroughly with 1× phosphate-buffered saline (PBS). All air bubbles were carefully removed, and coverslips were sealed with 4’,6-diamidino-2-phenylindole (DAPI) Fluoromount-G^®^ mounting medium for use as a nuclear counterstain and to provide a semi-permanent seal for storage of slide preparations. Images were visualized using a fluorescence microscope equipped with a MagnaFire Digital CCD Camera System (Eclipse TE300; Nikon, Tokyo, Japan).

#### 3.4.2. In Vitro Analysis of SIM NPs on the Formation of Mineralized Nodules

In our previous studies, the in vitro release SIM profile of SIM/PLGA-based nanoparticles showed that the burst release amount of the precipitated PLGA-based nanoparticles after dialysis in the colloid solution on the first day is still near 15–20% [20,21]. This SIM/APP nanoparticle system has a similar initial burst situation. Therefore, we directly use the fresh colloid solution after 4 h of water dialysis to avoid drug loss when we conduct in vitro or in vivo research, such as the mineralization in the extracellular matrix of D1 cells and in vivo experiments on rat OP disease. Before evaluating the mineralization ability in vitro and OP treatment in vivo, it was necessary to confirm the encapsulation efficiency and drug loading of SIM for APP NPs and PP NPs. However, calibration curves of SIM were obtained over the concentration range of 10^−4^ μM to 10^−8^ μM. Then, a 300 μL fresh colloid sample after dialysis (MWCO = 1000 Da, Spectro/Pro, Spectrum Lab. Inc. Irving, TX, USA) in distilled water for 4 h was treated with 500 μL of ethanol to dissolve SIM/APP and SIM/PP. Finally, 80 μL of the clear solution that passed through a 0.22 μm syringe filter was used for HPLC analysis (Lachrom Elite high-performance liquid chromatograph, Hitachi, Japan). The mobile phase consisted of 1.0% acetic acid in MeCN/methanol/water (45/30/25), and the flow rate was set at 1 mL/min. The absorbance of the column effluent was detected at 227 nm with a UV/VIS detector. Separation was achieved using an Inersil^®^ C18 (250 mm × 4.6 mm, 5 μm) analytical column connected to an Inersil^®^ C18 (50 mm × 4.6 mm, 5 μm) guard column. The column temperature was 40 °C. The weight of SIM within the APP NPs or PP NPs was calculated from the concentration of sample × volume of sample ((300 + 500 + 800 + 600) μL) × molecular mass of SIM. The encapsulation efficiency (EE%) was calculated by Formula (1), and the drug loading (DL) capacity was calculated by Formula (2).

(1) $$\mathrm{Encapsulation}\;\mathrm{efficiency}\;\left(\%\right)=\left(\mathrm{encapsulated}\;\mathrm{SIM}/\mathrm{feeding}\;\mathrm{SIM}\right)\times 100\%$$

(2) $$\mathrm{Drug}\;\mathrm{loading}\;\mathrm{capacity}\;\left(g/mg\right)=\mathrm{encapsulated}\;\mathrm{SIM}/\mathrm{total}\;\mathrm{weight}\;\mathrm{of}\;\mathrm{NPs}$$

Subsequently, Alizarin red S staining was used to determine the level of mineralization in the extracellular matrix, according to Tai et al.’s report [36]. Briefly, D1 cells were seeded into a 24-well plate at a density of 1 × 10^5^ cells/well. The cells were treated with different groups (blank, 0.5 μM SIM; equivalence, 0.5 μM SIM from fresh as-received SIM/APP NPs and SIM/PP NPs) in a culture medium for 5 days and then replaced with an osteoinduction medium for 1 additional day. The cells were fixed in 10% formalin and phosphate-buffered saline for 10 min. After washing twice with doubly distilled (dd) water, the fixed cells were stained with Alizarin red S solution for 5 min. After staining, the cells were washed using dd water. The fixed and stained plates were then air-dried at room temperature. The mineralization level was determined by dissolving the cell-bound Alizarin red S in 10% acetic acid and measuring it at 405 nm by using an ELISA reader (Synergy H1 Hybrid Multi-Mode Reader, BioTek, Winooski, VT, USA).

### 3.5. In Vivo Distribution Assay and OP Treatment Models in Rats

#### 3.5.1. In Vivo Distribution Assay of GNC-PLGA/APP NPs and GNC-PLGA/PP NPs in Rats

The biodistribution of the bone-targeting abilities of APP NPs and PP NPs should be re-evaluated because the GNC fluorescent contrast agent is different from the previous agents (IR-780) used in this study. Both NP systems were encapsulated with near-infrared (NIR) GNC-PLGA and evaluated in vivo in SD rats (purchased from BioLASCo Taiwan Co., Ltd.). In short, six 8-week-old SD rats were divided into two groups: one group was treated with bone-targeting GNC-PLGA/APP NPs, and the other group was treated with non-bone-targeting GNC-PLGA/PP NPs. For each experiment, 1.0 mL of NP colloids was injected into the tail vein of each rat. After 6 h, the animals were sacrificed, and the fluorescence signals (430–820 nm) were collected and quantified from major organs (such as heart, liver, spleen, lung, kidney) and bone (bilateral femur/tibia, and spine) using in vivo imaging systems (IVIS 200 Imaging System, Caliper Life Science, Alameda, CA, USA).

#### 3.5.2. In Vivo Experiments with Rat OP Models

Preoperative preparation and the surgical procedure were divided into two rat OP models and discussed briefly as follows:

#### 3.5.3. Postmenopausal OP

Twelve 8-week-old female SD rats were used to investigate the effects of bone-targeting drug nanocarriers (SIM/APP NPs) and non-bone-targeting drug nanocarriers (SIM/PP NPs) on postmenopausal OP (ovariectomized; OVX). They were housed individually in standard polycarbonate cages in a temperature-controlled room with a 12 h light and dark cycle and were provided standard rodent chow and water ad libitum. The rats were randomly assigned to four groups of three animals per group as follows: (1) sham group, (2) OVX only group, (3) fresh as-received SIM/PP NPs carrying SIM at a dose of 0.25 mg/kg per time, and (4) fresh as-received SIM/APP NPs also carrying SIM at a dose of 0.25 mg/kg per time. All rats were OVX and were left untreated for one month to allow significant osteopenia to develop. For OVX rats, bilateral dorsal incisions were made on the back of the rat, and both ovaries were identified. The blood vessels were clamped and tied off, and the ovaries were removed. The muscle layer was tied, and the skin incision was closed with silk sutures. The injection of NPs carrying SIM started one month after ovariectomization, and SIM was injected at a concentration of 0.25 mg/kg per injection 2 times per week for 2 months. All rats were sacrificed after 3 months of ovariectomization. Then, the distal portion of the tibia was scanned with a spatial resolution of 35 μm to quantify the bone mineral density (BMD, bone volume/total volume) using a high-resolution micro CT scanner (Skyscan 1076, Belgium Bruker, Billerica, MA, USA).

#### 3.5.4. Disuse OP

Twelve 8-week-old male Sprague–Dawley rats were used to investigate the effects of bone-targeting drug nanocarriers (SIM/APP NPs) and non-bone-targeting drug nanocarriers (SIM/PP NPs) on OP in a disuse environment. These SD rats were housed individually in 18 × 18 × 24 (L × W × H) acrylic cages designed by Dr. Marjolein van der Meulen (Cornell University) in a temperature-controlled room with a 12 h light and dark cycle and were provided with standard rodent chow and water ad libitum. The rats were randomly assigned into four groups of three animals per group as follows: (1) normal rats without hindlimb suspension, (2) hindlimb suspension rats without injection of SIM NPs, (3) hindlimb suspension rats injected with fresh as-received SIM/PP NPs carrying SIM at a dose of 0.25 mg/kg per time, and (4) hindlimb suspension rats injected with fresh as-received SIM/APP NPs also carrying SIM at a dose of 0.25 mg/kg per time.

Functional disuse was induced by the hindlimb suspension method [65]. Briefly, a nylon rope was attached to the tail with a piece of surgical tape, and the rope was attached to allow suspension from the top of the cage. An approximately 30° head-down tilt was set to prevent contact of the animal’s hindlimbs with the cage bottom. The animal’s forelimbs were allowed full access to the entire cage bottom. The injection of SIM NPs started 2 days after the hindlimb suspension, and SIM was injected at a concentration of 0.25 mg/kg per injection 2 times per week for 3 weeks. All rats returned to normal life one week after the 3-week hindlimb suspension. Finally, all rats were sacrificed, and the distal portion of the tibia was also scanned with a spatial resolution of 35 μm to quantitate the bone volume to total volume (BV/TV ratio) using a high-resolution micro CT scanner (Skyscan 1076, Bruker, Billerica, MA, USA).

### 3.6. Histological Analysis of Bone Tissue

Histochemical staining analyses of the distal portion of the tibia were also used to investigate the microscopic changes in the bone tissue for all of the above in vivo experiments with rats. Before hematoxylin–eosin staining, the bone tissue samples were decalcified (10% formic acid), followed by fixation with 4% paraformaldehyde. These samples were embedded in paraffin wax, and 5 μm sections were prepared. These sections were stained with hematoxylin–eosin.

### 3.7. Statistical Analysis

Data are presented as the mean ± SD of triplicate measurements unless noted otherwise. Student’s *t*-test was used to analyze the significance level for paired and unpaired data (GraphPad Prism Software Inc., La Jolla, CA, USA). * *p* < 0.05, and ** *p* < 0.01 denote the levels of significance.

## 4. Conclusions

A novel nanotherapeutic agent to treat osteoporosis (OP) was designed, composed of PLGA and PEG biopolymer serving as a nanocarrier for beneficial systemic circulation, a dendritic oligopeptide containing three aspartic acid molecules (_d_Asp_3_) as a bone-targeting moiety, and SIM as an OP drug. The size and negative charge of the spherical-like SIM/APP NPs used for bone targeting are less than 200 nm and ≤10 mV, respectively, and they are expected to be distributed in the bone tissue of interest by tail vein injection. Flow cytometry and colocalization fluorescence microscopy were used to evaluate the cellular uptake pathway, and the results showed that the APP NPs demonstrated effective cell uptake behavior through clathrin-dependent endocytosis. The cell mineralization assay suggested that the SIM/APP NPs could induce higher degrees of D1 cell mineralization as well as the formation of mineralized nodules. The in vivo distribution assay suggested that the bone-targeting moiety of dendritic oligopeptides composed of three aspartic acid molecules (_d_Asp_3_) in APP NPs could target bone, and the expected reduced side effects in other organs were affected by SIM. However, in vivo experiments were performed to compare the systemic bone loss of bilateral OVX (postmenopausal model) and the local bone loss of hindlimb suspension (disuse model) in a related low-dose SIM treatment (0.25 mg/kg per time, 2 times per week) for both kinds of OP rat models. The results showed that the bone-targeting SIM/APP NPs could increase the bone formation effect compared with non-bone-targeting SIM/PP NPs in the disuse model, while in the postmenopausal model for systemic OP, bone formation was not obvious. In vivo OP model testing showed that the same dose could not be sued to treat different types of OP, indirectly demonstrating that the different causes of OP require different treatment strategies. The bone-targeting moiety of dendritic oligopeptides composed of three aspartic acid (_d_Asp_3_) and functional PEG-PLGA NPs could effectively target bone and reduce the side effects in other organs affected by SIM. In other words, these bone-targeting SIM/APP NPs can accumulate to an effective SIM concentration in bone tissue with a high hydroxyapatite content, which will open a new avenue for the treatment of OP.

## Figures and Tables

**Table 1:** Size distribution analysis of PLGA-PEG-OMe (PP) NPs, GNC-PLGA/PP NPs, _d_Asp_3_-PEG-PLGA (APP) NPs, and GNC-PLGA/APP NPs was evaluated by dynamic light scattering (DLS) analysis.

Nanoparticles (NPs)	APP	GNC-PLGA/APP	PP	GNC-PLGA/PP
Average size (nm)	213.5	247.8	130.5	225.5
Polydispersity index (PDI)	0.116	0.029	0.024	0.005

**Table 2:** The size distribution, zeta potential, encapsulation efficiency (EE%), and drug loading (DL) capacity of the simvastatin nanocarriers were measured by dynamic light scattering (DLS), zeta potential analysis, and high-performance liquid chromatography (HPLC).

SIM Nanocarriers	Average Size (nm)	Polydispersity Index (PDI)	Zeta Potential (mV)	EE%	DL (μg/mg)
SIM/APP NPs	196.8	0.474	−35.5 ± 1.2	72.48%	0.28
SIM/PP NPs	103.8	0.121	−25.2 ± 0.5	76.29%	0.31

**Table 3:** In vivo investigations used systemic delivery of SIM and SIM nanocarriers in experimental models relevant to osteoporosis or osteogenesis.

Animal Model	Treatment Strategy	Dose and Duration	Main Findings	Reference
Rat [OVX]	Simvastatin (PO)	0.3 to 10 mg/kg per day for 60 days	Simvastatin had no role in new bone formation and resorption.	Yao et al., 2006 [57]
Rat [OVX]	Simvastatin (PO)	10 and 20 mg/kg per day for 42 days	Simvastatin increased bone volume, osteoblast number, BMP-2, collagen type I, and osteocalcin.	Ho et al., 2009 [58]
Rat [OVX]	Simvastatin (per oral) + 17-b-estradiol (IP)	5 or 10 mg/kg per day for 42 days	Simvastatin improved lumbar vertebral bone mineral density and mechanical properties. Simvastatin exerted opposing modulatory effects on estrogen receptor-alpha (Erα) expression on bone and uterus in ovariectomized rats.	Li et al., 2011 [59]
Rat [OVX]	SIM-loaded TC-PLGA NPs (IV)	0.5 mg/kg/2 days for 60 days	The SIM-loaded TC-PLGA NPs can improve the curative effects of SIM on the recovery of bone mineral density compared to either SIM-loaded PLGA NPs or SIM alone.	Wang et al., 2015 [29]
Rat [OVX]	SIM/ASP_6_-LNPs (IV)	1 mg/kg per 2 days for 60 days	SIM/ASP_6_-LNP nanocarriers can significantly improve the efficacy of SIM in the recovery of bone mineral density when compared with either SIM/LNPs or SIM alone.	Tao et al., 2020 [30]

Note: OVX: ovariectomized; IP: intraperitoneal; PO: oral gavage; IV: intravenous.

## Data Availability

Not applicable.

## Supplementary Materials

The following supporting information can be downloaded at: www.mdpi.com/article/10.3390/ijms231810530/s1. Reference [66] is cited in the Supplementary Materials.
