# Supplementary material for: Bone-Targeting Nanoparticles of a Dendritic (Aspartic acid)3-Functionalized PEG-PLGA Biopolymer Encapsulating Simvastatin for the Treatment of Osteoporosis in Rat Models
Source: Int J Mol Sci. 2022 Sep 11;23(18):10530. doi: 10.3390/ijms231810530 (PMC9503052; doi:10.3390/ijms231810530)
Supplement: Supplementary file 1 [file ijms-23-10530-s001.zip › ijms-1878987-supplementary.pdf]

Supporting Information

Bone-targeting nanoparticles of a dendritic (aspartic acid)$_3$-functionalized PEG-PLGA biopolymer encapsulating simvastatin for the treatment of osteoporosis in rat models

Che-Wei Lin[1,2,3,+], Chih-Yun Lee[2,3,4,+], Sung-Yen Lin[2,3,5,6,7], Lin Kang[8], Yin-Chih Fu[2,3,5,6,7], Chung-Hwan Chen[2,3,5,6,7,9], and Chih-Kuang Wang[1,2,3,4,10,*]

[1] Department of Medicinal and Applied Chemistry, College of Life Science, Kaohsiung Medical University, Kaohsiung 80708, Taiwan
[2] Regenerative Medicine and Cell Therapy Research Center, Office of Research and Development, Kaohsiung Medical University, Kaohsiung 80708, Taiwan
[3] Orthopaedic Research Center, College of Medicine, Kaohsiung Medical University, Kaohsiung 80708, Taiwan
[4] Ph.D. program in Life Sciences, College of Life Science, Kaohsiung Medical University, Kaohsiung 80708, Taiwan
[5] Departments of Orthopaedics, School of Medicine, College of Medicine, Kaohsiung Medical University, Kaohsiung 80708, Taiwan
[6] Department of Orthopaedics, Kaohsiung Medical University Hospital, Kaohsiung Medical University, Kaohsiung 80708, Taiwan
[7] Department of Orthopaedics, Kaohsiung Municipal Ta-Tung Hospital, Kaohsiung Medical University, Kaohsiung 80145, Taiwan
[8] Department of Obstetrics and Gynecology, National Cheng Kung University Hospital, College of Medicine, National Cheng Kung University, Tainan 70101, Taiwan
[9] Ph.D. Program in Biomedical Engineering, College of Medicine, Kaohsiung Medical University, Kaohsiung 80708, Taiwan
[10] Graduate Institute of Medicine, College of Medicine, Kaohsiung Medical University, Kaohsiung 80708, Taiwan
* Correspondence: ckwang@kmu.edu.tw; Tel.: +886-7-312-1101 (ext. 2677)
† These authors contributed equally to this work.

The APP verification is briefly described, and the related chemical evidence for each step, comprising the ^{1}H NMR spectrum and ESI mass spectrum, is presented in Figure S1 in the Supporting Information. To characterize the $_d$Asp$_3$-(OMe)$_4$ moiety, the product was evaluated by ^{1}H NMR spectroscopy and ESI-MS, as shown in Figure S1(d) and 1(e). The ^{1}H NMR spectra revealed triplet peaks at ~3.74–3.84 ppm that correspond to OCH_3, a peak at ~3.29 ppm attributed to CH_2, and a peak at 4.65 ppm attributed to CH. The ESI-mass spectrum revealed a molecular weight of 420.20 Da, indicating that the synthesis of the $_d$Asp$_3$-(OMe)$_4$ moiety was successful. The following steps were used to synthesize $_d$Asp$_3$-PEG-NH$_2$ polymers and $_d$Asp$_3$-PEG-PLGA. $_d$Asp$_3$-(OMe)$_4$ reacted with heterobifunctional NH$_2$-PEG-COOH to form H$_2$N-PEG-$_d$Asp$_3$-(OMe)$_4$ by amide bond formation. The bone-targeting functional polymer H$_2$N-PEG-$_d$Asp$_3$ was obtained after removing OMe from H$_2$N-PEG-$_d$Asp$_3$-(OMe)$_4$, and relevant chemical evidence from the FTIR spectra is also shown in Figure S2(d) in the Supporting Information. First, the carboxylic groups of the aspartic acid side chains were methylated; i.e., the O=C-OH groups were converted to O=C-CH$_3$. The FTIR spectrum of $_d$Asp$_3$-(OMe)$_4$ revealed a peak at ~1729 cm^{-1}, which was attributed to O=C stretching. The FTIR spectrum of H$_2$N-PEG-COOH indicated antisymmetric stretching of the ether group (1095 cm^{-1}). However, H$_2$N-

PEG-COOH reacted with $_{d}Asp_3$-$(OMe)_4$, and peaks at ~1729 cm^{-1} and ~1095 cm^{-1} were also observed in the FTIR spectrum, indicating the presence of ester and amide bonds, respectively. The H_2N-PEG-$_{d}Asp_3$-$(OMe)_4$ copolymer indicated successful conjugation from H_2N-PEG-COOH and $_{d}Asp_3$. To complete the bone-targeting functional amphiphilic block copolymer $_{d}Asp_3$-PEG-PLGA, NH_2-PEG-$_{d}Asp_3$-$(OMe)_4$ lost its methoxy (OMe) moiety to form the bone-targeting functional segment NH_2-PEG-$_{d}Asp_3$. Then, NH_2-PEG-$_{d}Asp_3$ directly conjugated with PLGA-COOH through an amide linkage to form the amphiphilic block copolymer $_{d}Asp_3$-PEG-PLGA. The ^1H NMR spectra verified the formation of $_{d}Asp_3$-PEG-PLGA, as shown in Figure S2(e) in the Supporting Information. These spectra showed a signal peak at 1.56 ppm attributed to PLA methyl protons (CH_3), a peak at 4.66–4.81 ppm attributed to PGA methylene protons (CH_2), and a peak at 3.64 ppm attributed to PEG ether linkages. However, the ^1H-NMR signals of $_{d}Asp_3$ were much less intense than those of PLGA and PEG and were not visible. The FTIR and ^1H-NMR spectra confirmed that H_2N-PEG-$_{d}Asp_3$ was conjugated to the PLGA chain to form the bone-targeting functional amphiphilic block copolymer $_{d}Asp_3$-PEG-PLGA. The amphiphilic copolymer PLGA–PEG was synthesized by direct conjugation of PLGA– COOH with H_2N–PEG–OMe by an amide linkage. To characterize the PLGA–PEG copolymer, the product was evaluated by ^1H-NMR spectroscopy, as shown in Figure S3 (b), and a signal peak at 1.56 ppm attributed to PLA methyl protons (PLA CH_3), a peak at 4.66–4.81 ppm attributed to PGA methylene protons (CH_2), and a peak at 3.64 ppm attributed to PEG ether linkages were observed. Therefore, the ^1H-NMR spectra confirmed that H_2N–PEG–OMe was incorporated into the PLGA chain.

(a)

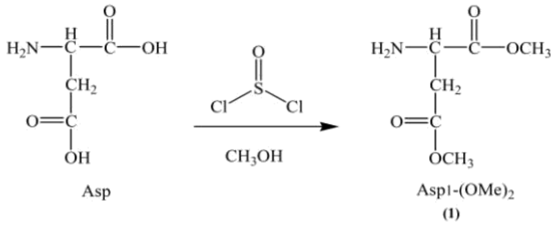
Asp
CH_3OH
Asp1-(OMe)$_2$
(1)

(b)

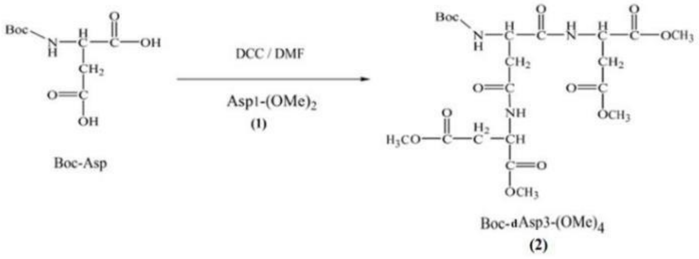
DCC / DMF
Asp1-(OMe)$_2$
(1)
Boc-Asp
Boc-dAsp3-(OMe)$_4$
(2)

(c)

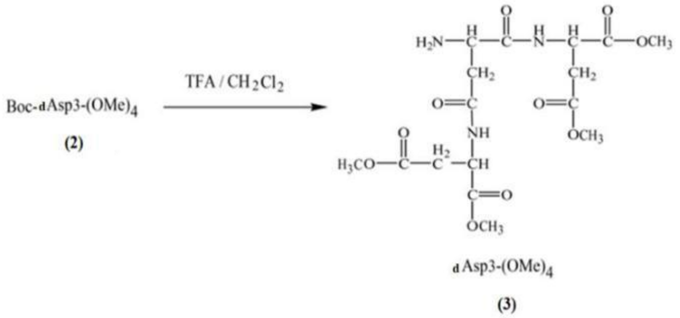
TFA / CH_2Cl_2
Boc-dAsp3-(OMe)$_4$
(2)
dAsp3-(OMe)$_4$
(3)

(d)

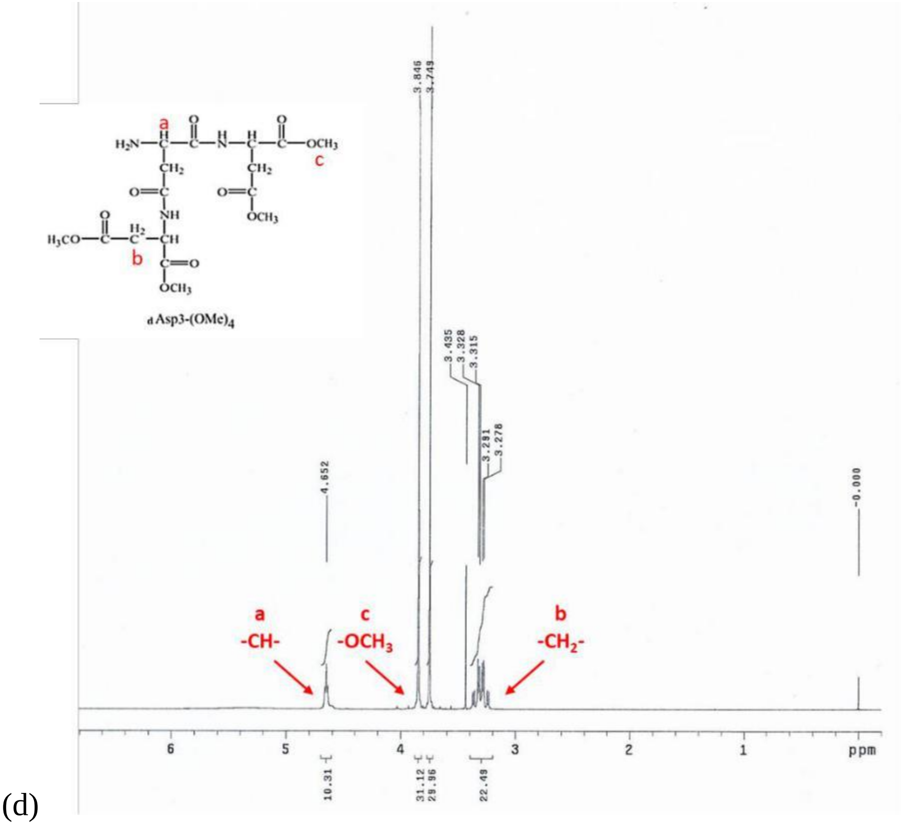
dAsp3-(OMe)$_4$
a
-CH-
c
-OCH$_3$
b
-CH$_2$-
4.652
3.846
3.749
3.435
3.328
3.315
3.291
3.278
0.000
ppm
10.31
31.12
29.96
22.48

(e) 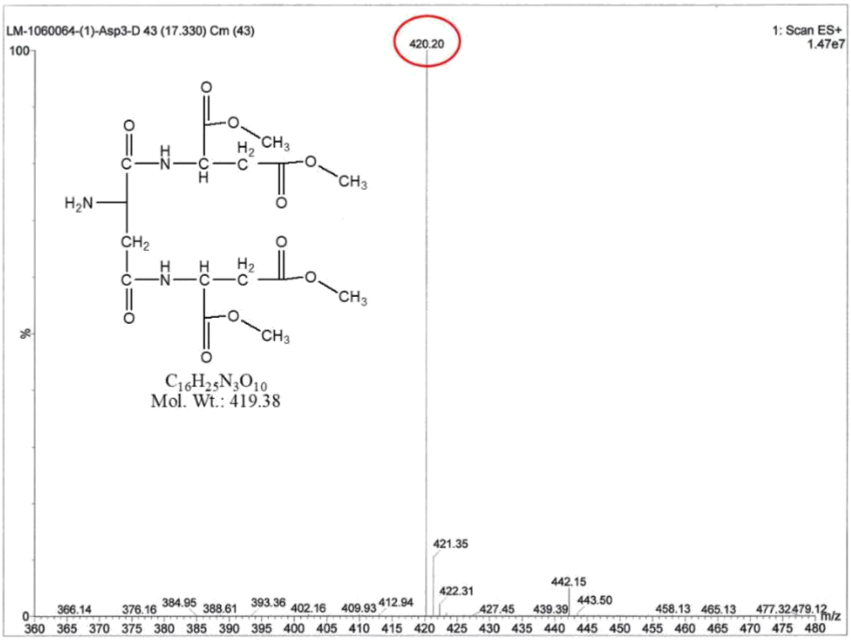

Figure S1. The synthesis mechanism and the steps used to make dAsp3-(OMe)4 moiety (a)~(c), which dAsp3 mean dendritic oligopeptide by three aspartic acid. The ^{1}H NMR spectra and ESI-Mass spectrum of the dAsp3-(OMe)4 were showed in (d) and (e).

(a)

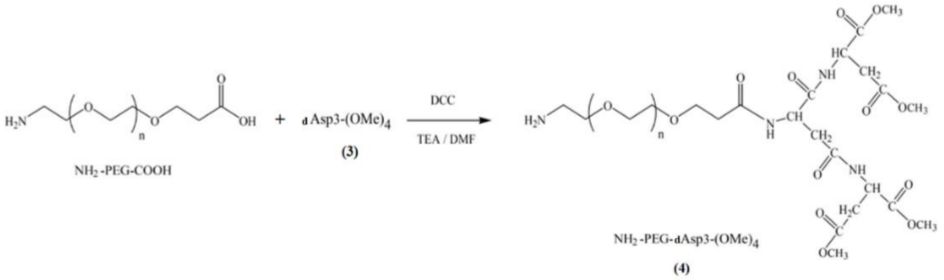

NH_2-PEG-COOH
dAsp3-$(OMe)_4$
(3)
DCC
TEA / DMF
NH_2-PEG-dAsp3-$(OMe)_4$
(4)

(b)

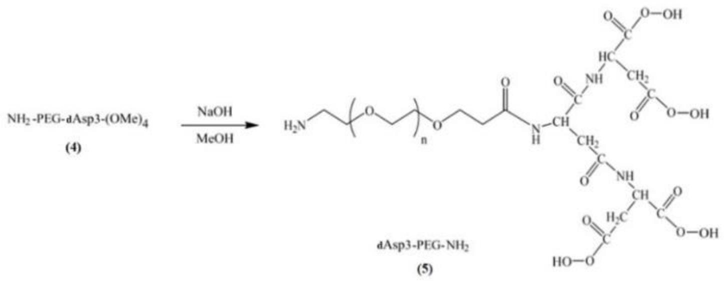

NH_2-PEG-dAsp3-$(OMe)_4$
(4)
NaOH
MeOH
dAsp3-PEG-NH_2
(5)

(c)

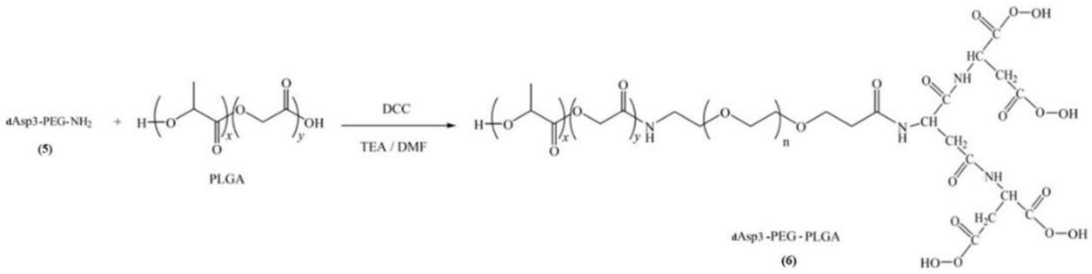

dAsp3-PEG-NH_2
(5)
PLGA
DCC
TEA / DMF
dAsp3-PEG-PLGA
(6)

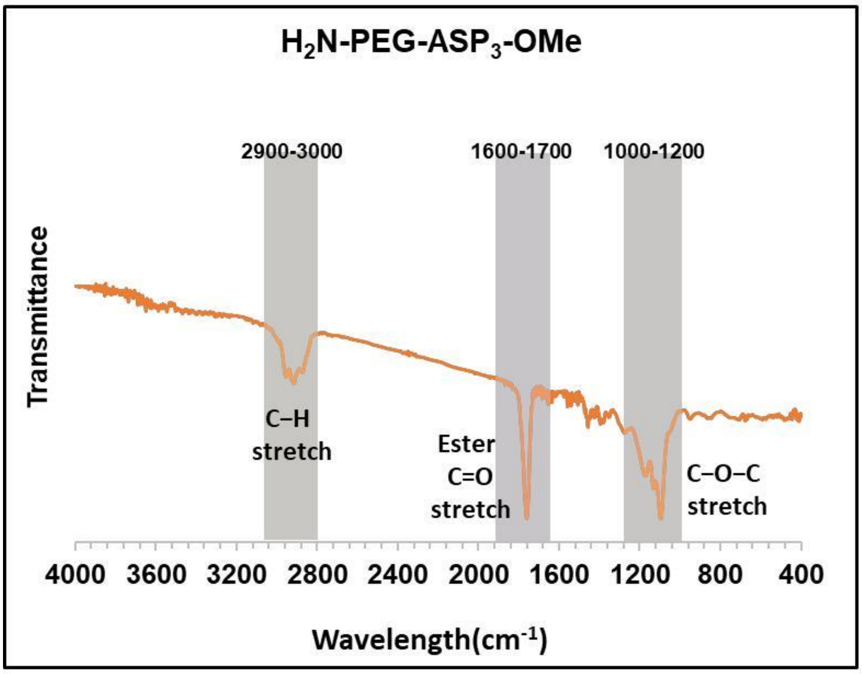

H_2N-PEG-ASP_3-OMe
2900-3000
1600-1700
1000-1200
Transmittance
C–H stretch
Ester C=O stretch
C–O–C stretch
4000 3600 3200 2800 2400 2000 1600 1200 800 400
Wavelength(cm^{-1})

(d)

(e)

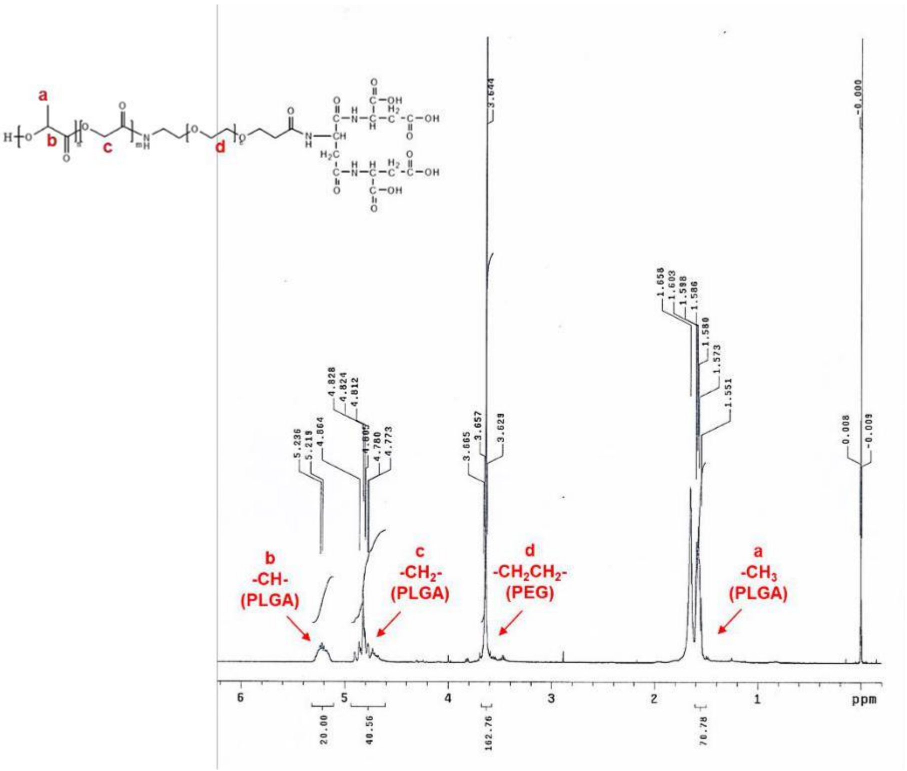

Figure S2. The synthesis mechanism and the steps used to make $_{\mathrm{d}}$Asp$_3$-PEG-NH$_2$ copolymers (a, b) and the $_{\mathrm{d}}$Asp$_3$-PEG-PLGA (c). However, the FTIR spectrum and the ^{1}H NMR spectra of the $_{\mathrm{d}}$Asp$_3$-PEG-PLGA (APP) copolymer were showed in (d) and (e).

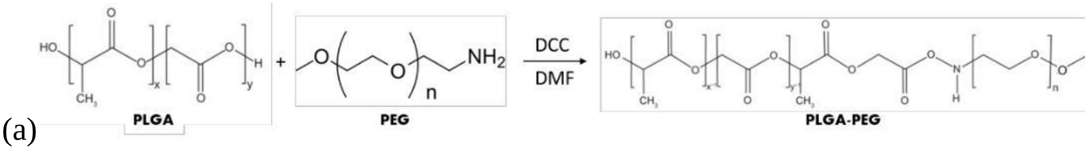

(a)

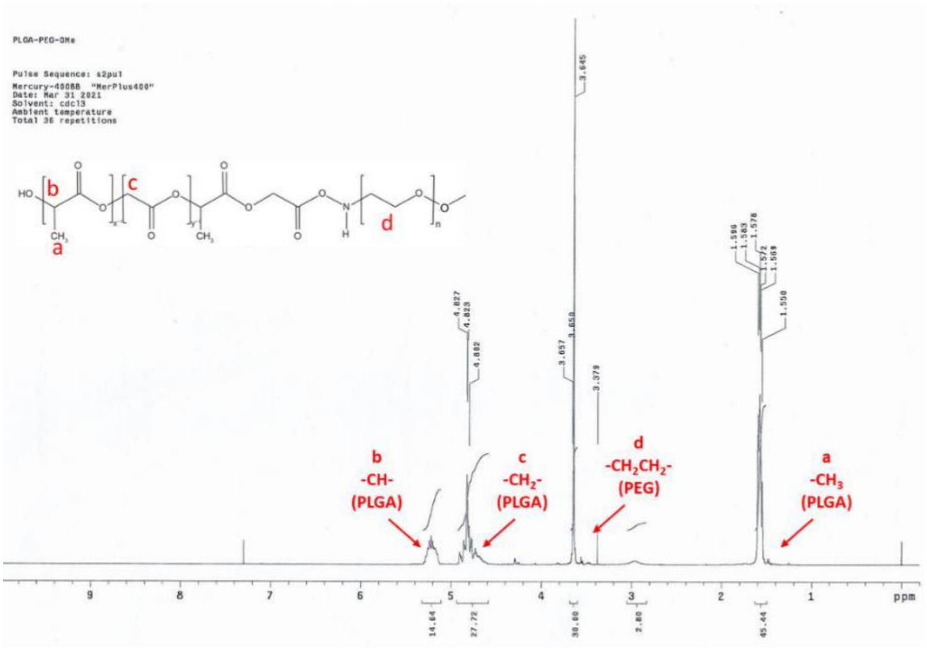

(b)

Figure S3. The synthesis scheme of the amphoteric block copolymer of PEG-PLGA (a) and ^{1}H NMR spectrum of PEG-PLGA (b).

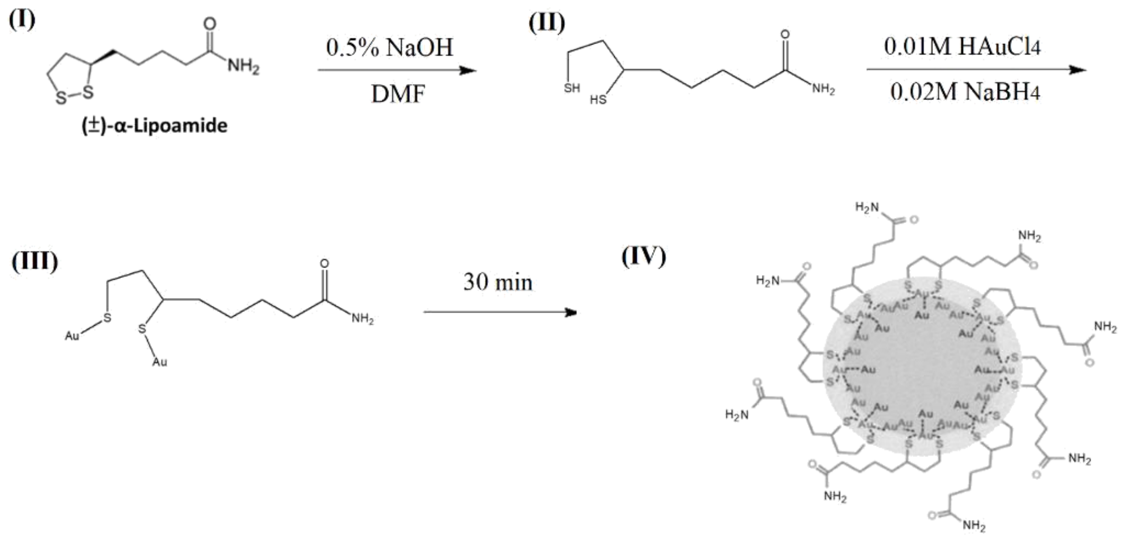

Figure S4. Schematic diagram of synthesis gold nanoclusters (GNCs) with 1° amide group using (±)-α-Lipoamide template.

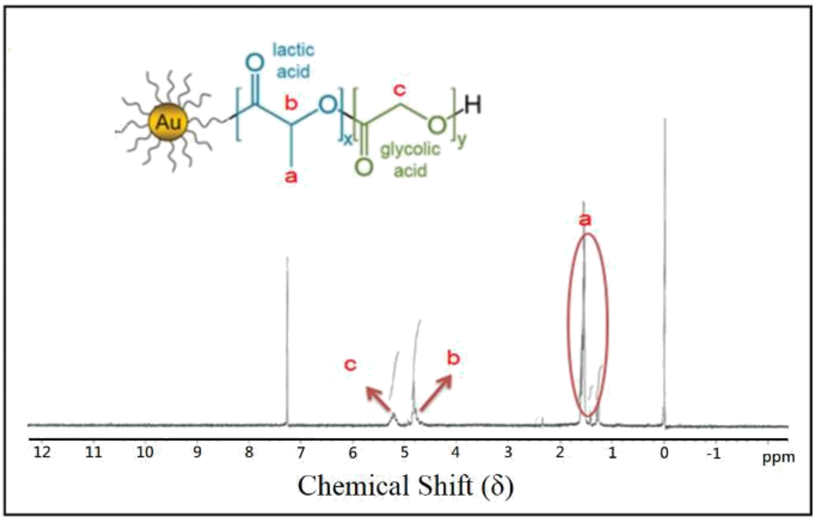

Figure S5. The ^{1}H NMR spectrum of *GNC* coupling with PLGA (GNC-PLGA).

The ^{1}H-NMR spectra verified the GNC-PLGA structure (Figure S5) showing a signal peak at 1.56 ppm representing PLA methyl protons (PLA CH_3), The peaks at 5.2 ppm, and 4.8 ppm are related to the CH-CH_3 from lactic acid and CH-H from glycolic acid, respectively. This confirms the synthesis of the PLGA copolymer [66]. There are no obvious GNC surface functional group peaks of lipoamide ligand since the amount of lipoamide functional group is too small. Whatever, the ^{1}H-NMR spectra also combined fluorescence spectrum (Fig. 2(c) of the manuscript) can be confirmed that GNCs were incorporated into the PLGA chain.

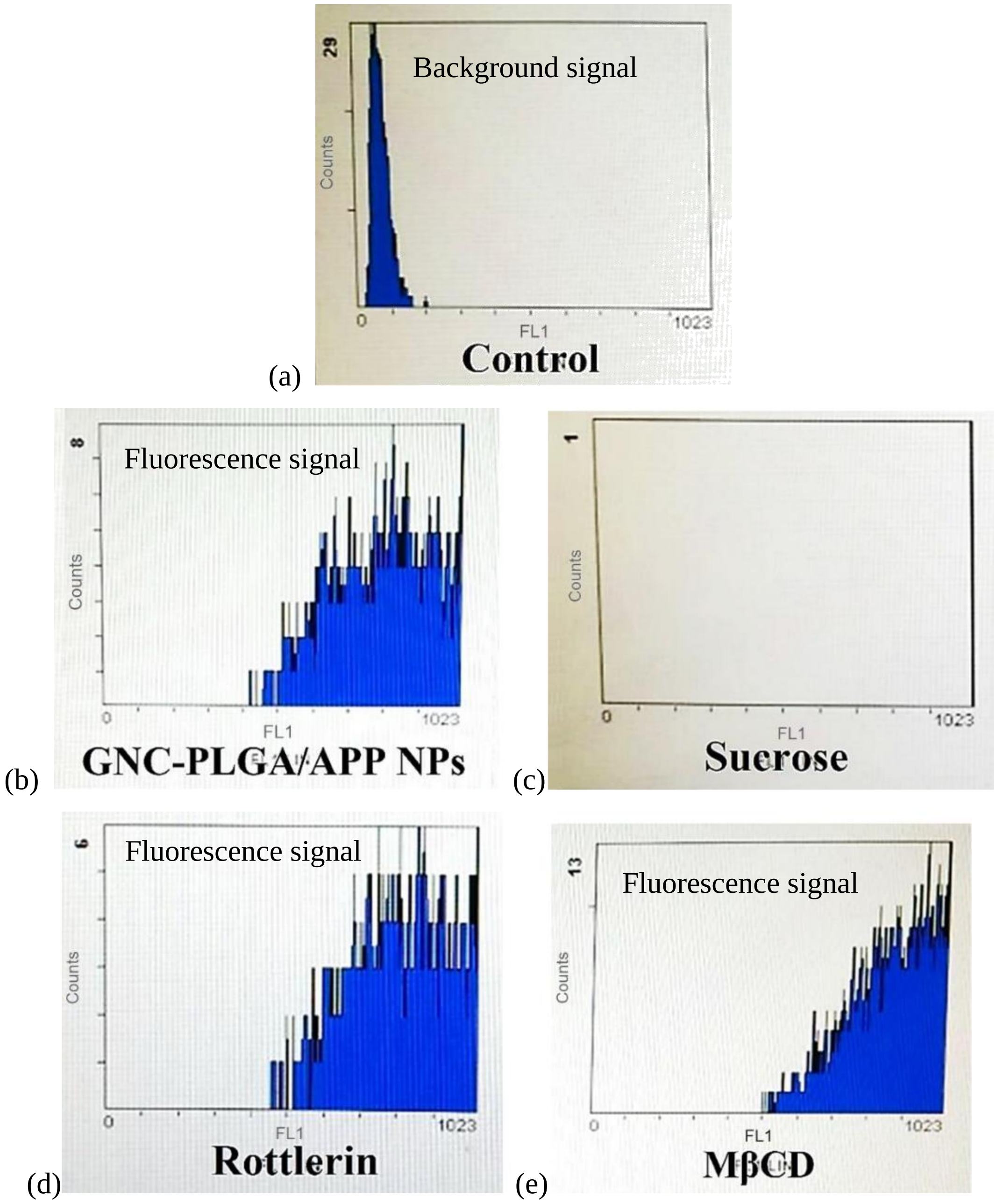

Figure S6. Side scattering intensity (SSC) histograms of D1 cells with incident light wavelengths. Typical flow cytometry results for untreated cells (a; Control) and cells exposed to fluorescently labeled GNC-PLGA/APP NPs (b~e). Flow cytometry-based quantitative analysis of the uptake of GNC-PLGA/APP NPs in D1 cells without inhibitors (b) and the presence of different endocytosis inhibitors, such as sucrose (c), Rottlerin (d), MβCD (e).

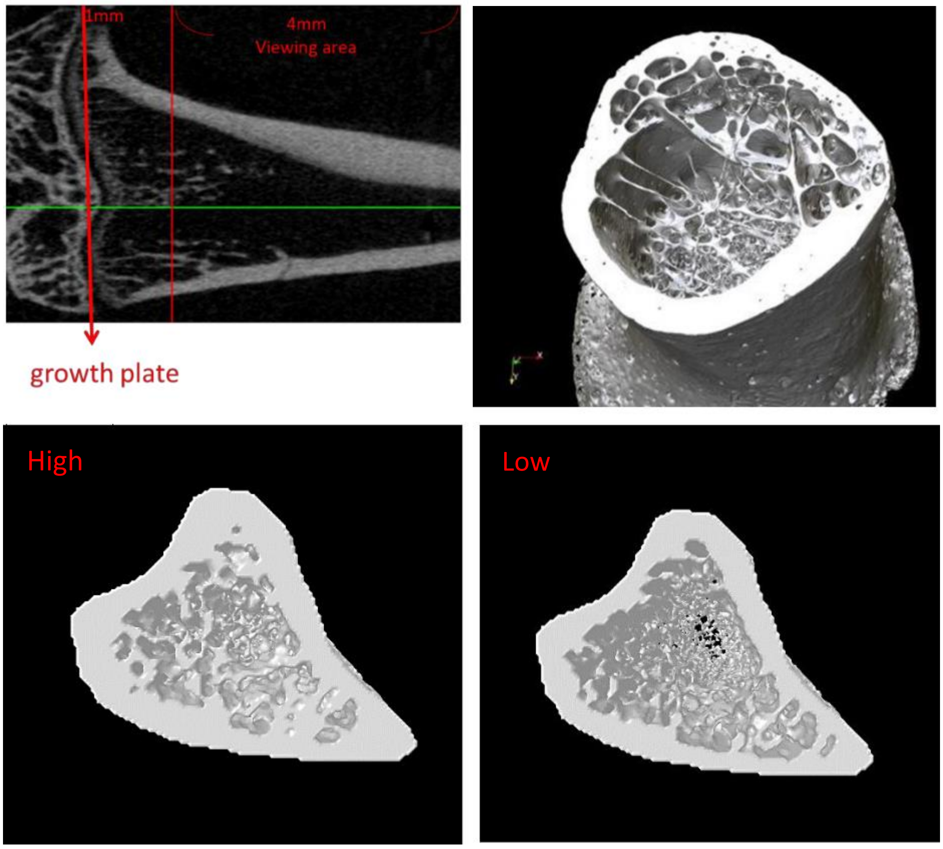

Figure S7. The distal portion of the tibia was scanned with a spatial resolution of 35 μm to reconstruct the 3D bone skeleton and quantitative bone volume to total volume (BV/TV ratio) by grayscale and using a high-resolution micro-CT scanner (Skyscan 1076, Belgium).

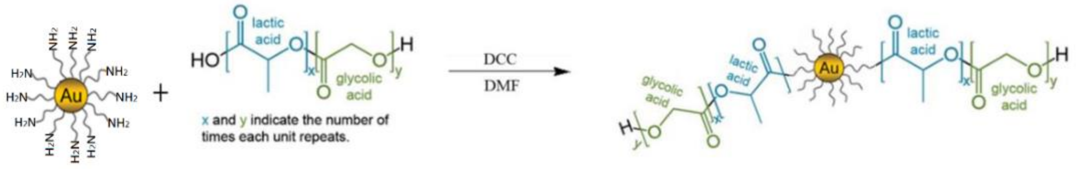

(a)

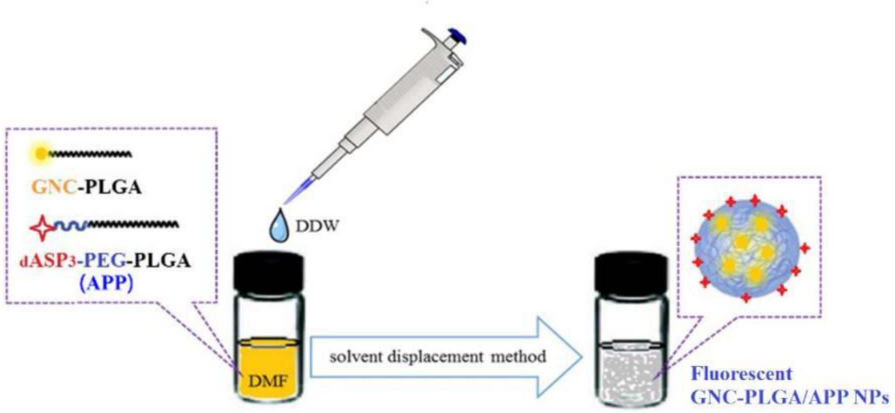

(b)

Figure S8. Schematic of the synthesis of gold nanocluster (GNC) coupled with PLGA (GNC-PLGA) (a) and the formation diagram of bone-targeted fluorescent nanoparticles composed of GNC-PLGA and dAsp3-PEG-PLGA (APP) block polymer, whose weight ratio is approximately 1/2 (b).
